# Analysis of retrotransposon abundance, diversity and distribution in holocentric *Eleocharis* (Cyperaceae) genomes

**DOI:** 10.1093/aob/mcy066

**Published:** 2018-05-04

**Authors:** Thaíssa B de Souza, Srinivasa R Chaluvadi, Lucas Johnen, André Marques, M Socorro González-Elizondo, Jeffrey L Bennetzen, André L L Vanzela

**Affiliations:** 1 Laboratory of Cytogenetics and Plant Diversity, Department of General Biology, Center for Biological Sciences, State University of Londrina, Londrina, Paraná, Brazil; 2 Department of Genetics, University of Georgia, Athens, GA, USA; 3 Laboratory of Genetic Resources, Campus Arapiraca, Federal University of Alagoas, Arapiraca, Brazil; 4 CIIDIR, Instituto Politécnico Nacional, Durango, Mexico

**Keywords:** *Copia*, DNA C-value, flow cytometry, *Gypsy*, holokinetic chromosomes, sedges, transposable elements

## Abstract

**Background and Aims:**

Long terminal repeat-retrotransposons (LTR-RTs) comprise a large portion of plant genomes, with massive repeat blocks distributed across the chromosomes. *Eleocharis* species have holocentric chromosomes, and show a positive correlation between chromosome numbers and the amount of nuclear DNA. To evaluate the role of LTR-RTs in karyotype diversity in members of *Eleocharis* (subgenus *Eleocharis*), the occurrence and location of different members of the *Copia* and *Gypsy* superfamilies were compared, covering interspecific variations in ploidy levels (considering chromosome numbers), DNA C-values and chromosomal arrangements.

**Methods:**

The DNA C-value was estimated by flow cytometry. Genomes of *Eleocharis elegans* and *E. geniculata* were partially sequenced using Illumina MiSeq assemblies, which were a source for searching for conserved proteins of LTR-RTs. POL domains were used for recognition, comparing families and for probe production, considering different families of *Copia* and *Gypsy* superfamilies. Probes were obtained by PCR and used in fluorescence *in situ* hybridization (FISH) against chromosomes of seven *Eleocharis* species.

**Key Results:**

A positive correlation between ploidy levels and the amount of nuclear DNA was observed, but with significant variations between samples with the same ploidy levels, associated with repetitive DNA fractions. LTR-RTs were abundant in *E. elegans* and *E. geniculata* genomes, with a predominance of *Copia* Sirevirus and *Gypsy* Athila/Tat clades. FISH using LTR-RT probes exhibited scattered and clustered signals, but with differences in the chromosomal locations of *Copia* and *Gypsy*. The diversity in LTR-RT locations suggests that there is no typical chromosomal distribution pattern for retrotransposons in holocentric chromosomes, except the CRM family with signals distributed along chromatids.

**Conclusions:**

These data indicate independent fates for each LTR-RT family, including accumulation between and within chromosomes and genomes. Differential activity and small changes in LTR-RTs suggest a secondary role in nuclear DNA variation, when compared with ploidy changes.

## INTRODUCTION

The DNA C-value has been recognized as a relevant parameter for plant genome studies, especially in the context of genome evolution (Bennett and Leitch, 2011). The amount of DNA can change quickly in genomes, due to ploidy and repetitive DNA accumulation/elimination, often without any obvious taxonomic effect (Kellogg and Bennetzen, 2004; Lee and Kim, 2014). The repetitive DNA fraction can be recognized and systematically organized according to repeat structure or size, phylogenetic distribution/origin, sequence relatedness, mode of amplification and/or genomic distribution (Jurka *et al.*, 2007; Wicker *et al.*, 2007). Primary components of the repetitive DNA are the transposable elements (TEs), and they are particularly important because of their roles in both genome and gene functions (Lisch, 2013; Makarevitch *et al.*, 2015). The nature and genomic representativeness of these sequences can also be used as parameters in studies of chromosome biology and karyotype organization (Marques *et al.*, 2015; Santos *et al.*, 2015; Ribeiro *et al.*, 2016).

Transposable elements are the most abundant repetitive sequences in plant genomes (SanMiguel *et al.*, 1996; Jurka *et al.*, 2007; Heslop-Harrison and Schwarzacher, 2011). Their proportion in dicotyledonous plants has been seen to vary from >10 % of the genome in arabidopsis to approx. 62 % of retroelements in the genome of *Solanum lycopersicum* (Arabidopsis Genome Initiative, 2000; Paz *et al.*, 2017). In monocotyledonous plants, such as rice, sorghum and maize, TEs routinely range from 40 to 85 % of total DNA (Meyers *et al.*, 2001; Matsumoto *et al.*, 2005; Schnable *et al.*, 2009; Devos, 2010). TEs that transpose using RNA intermediates by a ‘copy-and-paste’ process (Class 1) are commonly called retroelements, and those that transpose in a ‘cut-and-paste’ process (Class 2) are called DNA transposons (Jurka *et al.*, 2007; Wicker *et al.*, 2007). Retroelements fall into three primary categories: long terminal repeat-retrotransposons (LTR-RTs), long interspersed nuclear elements (LINEs) and short interspersed nuclear elements (SINEs). The most abundant LTR-RTs are members of either the *Copia* or *Gypsy* superfamilies (Bennetzen, 2000; Heslop-Harrison and Schwarzacher, 2011; Grandbastien, 2015). LTR-RTs represent approx. 14 % of the *Rhynchospora pubera*, Cyperaceae (Marques *et al.*, 2015) genome, but approx. 24 % and approx. 75 % of the sorghum (Paterson *et al.*, 2009) and maize (Schnable *et al.*, 2009) genomes, respectively. DNA transposons contribute quantitatively less to the genomes of these species, at approx. 8.8, 7.5 and 9 %, respectively.

Different TE families, defined by their different vertical origins, can occur either in clusters or scattered along chromosomes (see Gaeta *et al.*, 2010). In some species, some *Gypsy* and *Copia* families have been found to cluter in specific chromosomal regions. Centromeric retrotransposons (CRs) of the *Gypsy* superfamily, for example, preferentially accumulate at primary constrictions in monocentric chromosomes (Neumann *et al.*, 2011) and near centromeric protein-binding sites along holocentric chromatids (Marques *et al.*, 2015). Juncaceae and Cyperaceae are two families of monocotyledonous plants in which holocentric chromosomes are a synapomorphy (Greilhuber, 1995). This feature enables the parallel movement of chromatids during cell divisions in mitosis, differently from Rabl’s organization in monocentric chromosomes (Guerra *et al.*, 2010; Marques and Pedrosa-Harand, 2016), as well as the permanence of chromosome fragments generated by chromosome fission and fusion in subsequent cell divisions (Vanzela and Colaço, 2002; Hipp *et al.*, 2013). Information on LTR-RT distribution in plants with holocentric chromosomes is scarce. One such study shows, for example, that *Copia* and *Gypsy* retrotransposons appear as few and scattered signals on the chromosomes of *Luzula elegans*, a Juncaceae (Heckmann *et al.*, 2013). An exception is the complete description of co-localization of CRRh (centromeric retrotransposon of *Rhynchospora*), Tyba satellite DNA (satDNA; 172 bp length) and CENH3 proteins in holocentrics of *Rhynchospora* Cyperaceae (Marques *et al.*, 2015).

Holocentric chromosomes can be very tolerant of dysploidy events, and this can be observed in the wide chromosome number variation of the Cyperaceae (Da Silva *et al.*, 2008; Roalson, 2008; Bureš and Zedek, 2014). Species of *Eleocharis* R. Br. (Cyperaceae) exhibit variations in holocentric chromosome numbers from 2*n* = 6 to 2*n* = 196, including dysploids and polyploids (Hoshino, 1987; Da Silva *et al.*, 2005, 2010; Roalson, 2008). They also exhibit variation in the amount of DNA, ranging from 18 pg in *E. sterneri* to 0.84 pg in *E. cellulosa* (Zedek *et al.*, 2010). In order to shed light on the organization of TEs in these holocentric chromosomes, we used low coverage DNA sequencing and fluorescence *in situ* hybridization (FISH) to investigate *Eleocharis* TE families for their possible distribution variance related to karyotype structure and DNA C-value. Partial assemblies of *E. elegans* and *E. geniculata* genomes were used as a database for TE discovery and FISH probe design. These experiments investigated differences in LTR-RT family accumulations and distributions, with a special focus on identifying family predominance, hotspots for co-localization and effects on karyotype diversity.

## MATERIALS AND METHODS

### Plant materials

A minimum of five individuals from seven *Eleocharis* species belonging to *Eleocharis* subgenus *Eleocharis* [*E. maculosa* and *E. geniculata* (two populations each), *E. elegans*, *E. sellowiana*, *E. filiculmis*, *E. montana* and *E. niederleinii*] were selected because of their known karyotype diversity (Da Silva *et al.*, 2008, 2010), and collected in the South and South-east of Brazil (Table 1). Plants were grown in pots in the greenhouse of the Center for Biological Sciences at the State University of Londrina, and vouchers (Table 1) were deposited in the herbarium at FUEL, Brazil.

**Table 1.** Information on chromosome numbers, ploidy levels and nuclear DNA content for *Eleocharis* species, and three species used as controls

Species	2*n*	NAN	Mbp (2C)	Pg ± s.d.	pg (C*x*)	HSD	Vouchers
*E. maculosa* (Vahl) Roem. & Schult.	2*n* = 2*x* = 6	40 487	841.11	0.86 ± 0.04	0.43	a	55228
	2*n* = 2*x* = 10	56 342	976.42	1.00 ± 0.04	0.49	c	55227
*E. geniculata* (L.) Roem. & Schult.	2*n* = 2*x* = 10	45 661	985.55	1.01 ± 0.03	0.50	c	CRMS-2
	2*n* = 4*x* = 20	49 597	1814.12	1.85 ± 0.08	0.46	b	55225
*E. sellowiana* (Poir.) Urb.	2*n* = 4*x* = 20	50 048	1608.46	1.64 ± 0.07	0.41	a	55226
*E. elegans* (Kunth)	2*n* = 4*x* = 20	34 609	2285.19	2.34 ± 0.11	0.58	e	55224
*E. filiculmis* (Kunth)	2*n* = 6*x* = 30	97 343	3105.15	3.18 ± 0.05	0.53	d	55226
*E. montana* (L.) Roem. & Schult.	2*n* = 8*x* = 40	72 090	4584.27	4.69 ± 0.09	0.58	e	55223
*E. niederleinii* Boeckeler	2*n* = 8*x* = 42	40 887	4918.74	5.03 ± 0.20	0.63	f	55343
*R. breviuscula* H. Pfeiff	2*n* = 10	>30 000	782.40	0.80	–	–	–
*R. pubera* (Vahl) Boeckeler	2*n* = 10	>30 000	3217.62	3.29	–	–	–
*S. lycopersicum* L. ‘Stupické polní rané’	2*n* = 24	>30 000	1916.88	1.96	–	–	*

2*n*, diploid chromosome number; NAN, number of analysed nuclei; Mbp (2C), C-value in somatic cells in mega base pairs; pg ± s.d., average picogram value for 2C ± s.d.; pg (C*x*), value in picograms for each monoploid complement; HSD (honestly significant difference), mean comparisons test, where different letters differ significantly from each other, using Tukey’s test at 5 % probability;

*Seeds supplied by Jaroslav Doležel, Department of Cell Biology and Genetics, Palacky University, Czech Republic.

### Genome size estimation and chromosome number counts

Measurements of DNA C-values were done with young culms using 1 mL of cold LB01 buffer plus 1 mg mL^–1^ propidium iodide (Life Technologies), according to Doležel *et al.* (2007). Analyses were performed on a BD ACCURI C6 flow cytometer, in three independent estimations on different days. *Solanum lycopersicum* ‘Stupické polní rané’ (2C = 1.96 pg), as well as *Rhynchospora pubera* (2C = 3.53 pg) and *R. breviuscula* (2C = 0.80 pg) were used as standards (values from Doležel *et al.*, 2007; Marques *et al.*, 2015; Rocha *et al.*, 2016, respectively). The 2C values were calculated as sample peak mean/standard peak means × 2C DNA amount of standard (pg). Analyses of variance (ANOVAs) were performed using the Sisvar 5.6 program, considering monoploid complement values per sample. The means comparison (Tukey’s HSD, <5 %) and the Pearson correlation tests were performed in the program R (/www.r-project.org), and the graphs were elaborated with the Gnumeric program (Linux).

Mitotic chromosomes were obtained from root tips treated with 2 mm 8-hydroxyquinoline for 24 h, and fixed in a fresh solution of ethanol:acetic acid (3:1, v/v) for 24 h. Samples were softened in 2 % cellulase and 20 % pectinase (w/v) at 37 °C for 1 h, hydrolysed in 1 m HCl for 10 min at 60 °C, and squashed in a drop of 60 % acetic acid. After removal of the coverslip using freezing in liquid nitrogen, slides were stained in 2 % Giemsa and mounted with Entellan (Merck). Chromosome counts were performed in at least ten cells for each sample.

### *In silico* analyses

A small quantity of genome sequence data for *E. elegans* (NCBI: SRX3256858) and *E. geniculata* (NCBI: SRX3256859) was generated using Illumina Miseq PE250. The assemblies were done with the SPAdes program considering three different K-mers, and with input files containing 5 448 113 and 1 177 302 reads, respectively (Supplementary Data Table S1). For screening the assembled sequences containing conserved stretches of TEs, sequences >150 bp in length were compared against RepBase (http://www.girinst.org/censor/) and GypsyDB (http://gydb.org/index.php/MainPage) databases. A second screening was done for conserved gag-POL regions using a local BLASTx run against a custom database containing the main families of plant TEs, grouped according to classes and families (Llorens *et al.*, 2009): Class 1 LTR-RTs: *Gypsy* (Reina, Galadriel, Del, CRM, Athila and Tat) and *Copia* (Oryco, Retrofit, SIRE and Tork), and Class 2 transposons: CACTA, *Mutator*, Harbinger, hAT and Helitrons.

Because the databases do not have reference sequences from Cyperaceae, we searched for an initial cut of 60 % identity (E-value 10e-4) for conserved protein regions of gag-POL, transposase and helicase. Sequences with ≥80 % identity for integrase, RNase H and reverse transcriptase of both *Copia* and *Gypsy* members were used as templates for primer design, using the OligoPerfect™ Designer tool (http://tools.lifetech nologies.com).

The similarity tree was based on reverse transcriptase fragments selected after local BLASTx, comparing *E. elegans* and *E. geniculata* sequences against GypsyDB sequences, according to E-value 10e-4, >80 % identity and >300 bp in length. Reverse transcriptase sequences were analysed with SATé-v2.2.7 software for alignment (MAFFT and MUSCLE) and development of a maximum likelihood tree (FASTTREE) that was edited using Figtree v1.4.2.

The GC content of the assemblies and of the TE fractions was estimated from the *E. elegans* and *E. geniculata* genomes using the Illumina R1 and R2 output files with the FastQC tool. Estimates were also done after SPAdes assembling, and after screening using the TE portions against the RepBase database. For this, investigations of GC vs. AT content were done with SED/GREP pipelines (Supplementary Data Table S3).

### DNA extraction and PCR

DNA was isolated from young culms of seedlings from each species that were macerated in liquid nitrogen and treated with 2 % cetyltrimethylammonium bromide (CTAB) extraction buffer. DNA was purified with phenol:chloroform (1:1, v/v), chloroform:isoamyl alcohol (24:1, v/v) and RNase (1 mg mL^–1^), and precipitated in 100 % absolute ethanol. Ethanol-precipitated DNAs were resuspended in 10 mm Tris–HCl pH 8. DNA concentrations were estimated using a NanoDrop 2000 Spectrophotometer (Thermo Scientific).

The LTR-RT probes were obtained by PCR using specific primers for each family with each one of the seven species as template DNA (Supplementary Data Table S2). A standard PCR [5 U µL^–1^*Taq* polymerase (0.5 µL), 10× buffer (2.5 µL), 50 mm MgCl_2_ (1.5 µL), 10 mm dNTP (1 µL), 5 mm primers (2 µL each), and H_2_O up to a final volume of 25 µL] was used in the following conditions: 94 ºC for 2 min, 30 cycles of 94 ºC for 40 s, 59 ºC for 40 s and 72 ºC for 1 min, and a final extension of 72 ºC for 10 min. Reactions were tested using electrophoresis in an agarose gel at 3 V cm^–1^ and stained with ethidium bromide. In the end, the reactions were found to be adequate for each primer set.

The PCR products were used in a second reaction to produce templates for Sanger sequencing, using the 3500×L Automatic Sequencer (Applied Biosystems), according to the manufacturer’s procedures. Two distinct reactions for each primer (forward and reverse) were carried out, and repeated once. The consensus sequences were obtained after alignment of these sequences, in which quality was tested with Phred/Phrap/Consed software, and these sequences were compared against GenBank (http://www.ncbi.nlm.nih.gov/blast) and against POL conserved protein cores available in the GypsyDB.

### Fluorescence *in situ* hybridization

Fluorescence *in situ* hybridization was performed as described by Da Silva *et al.* (2008). Slides were prepared by squashing without acid hydrolysis. Probes for each LTR-RT family were obtained using a reamplification of PCR products that involved labelling with biotin-11-dUTP (*Copia* families) or Cy3-dUTP (*Gypsy* families). Each probe was mixed with a solution (30 μL) composed of 100 % formamide (15 μL), 50 % polyethylene glycol (6 μL), 20× SSC (3 μL), 100 ng of calf thymus DNA (1 μL), 10 % SDS (1 μL) and 100 ng of probes (4 μL). The mix was denatured at 90 °C for 10 min, and hybridization was performed at 37 °C during 24 h in a humid chamber. Post-hybridization washes were carried out with 70 % stringency using SSC buffer, pH 7.0. After probe detection with avidin–fluorescein isothiocyanate (FITC) conjugate, washes were performed in 4× SSC/0.2 % Tween-20, all at room temperature, and slides were mounted with 25 μL of DABCO, a solution composed of glycerol (90 %), 1,4-diaza-bicyclo (2.2.2)-octane (2.3 %), 20 mm Tris–HCl, pH 8.0 (2 %), 2.5 mm MgCl_2_ (4 %) and distilled water (1.7 %), plus 1 μL of 2 μg mL^–1^ 4,6’-diamidino-2-phenylindole (DAPI).

### Image acquisition

All chromosome images were acquired in grey-scale with a Leica DM4500 B microscope coupled with a DFC 300FX camera, and they were pseudo-coloured (blue for DAPI, greenish-yellow for FITC and red for Cy3) and contrasted, using GIMP 2.8 Linux.

## RESULTS

**Fig. 1.**
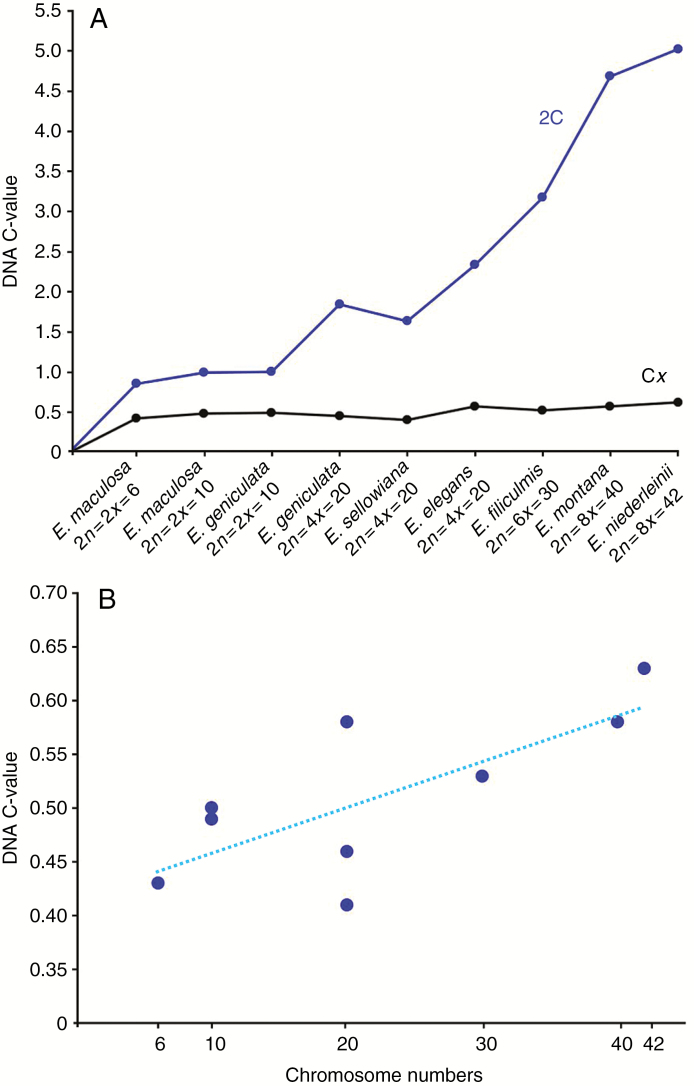
Correlation between chromosome number, ploidy levels and estimated amount of DNA for nine samples belonging to seven species of *Eleocharis*. (A) Comparison between 2C values (total DNA) and the probable monoploid complement (C*x*). Note that although the increase in DNA content accompanied the increase in the ploidy level, significant variations were observed between species with the same chromosome number, as well as in the dysploid species *E. maculosa* (2*n* = 6) and *E. niederleinii* (2*n* = 42). (B) Correlation between the chromosome number and DNA C-value, based on the monoploid complement (C*x*), showing *R*^2^ = 0.712

The amount of DNA (2C) measured using at least 30 000 nuclei per species [coefficient of varaiation (CV) <5 %] showed a range of 0.86 ± 0.04 pg in *E. maculosa* (2*n =* 2*x =* 6) to 5.03 ± 0.20 pg in *E. niederleinii* (2*n =* 8*x =* 42). The comparative analysis of monoploid complement values (C*x*) showed that typical diploids such as *E. maculosa* and *E. geniculata* (2*n* = 2*x* = 10) exhibited a C*x* of approx. 0.50 pg, while larger variations were observed among polyploids. *Eleocharis sellowiana* (2*n* = 4*x* = 20) exhibited the lowest C*x* value (0.41 pg), but *E. elegans* and *E. montana* presented different ploidy levels (2*n* = 4*x* = 20 and 2*n* = 8*x* = 40, respectively) with an average C*x* value of 0.58 pg (Table 1; Supplementary Data Fig. S1). Other polyploid species, such as *E. geniculata* with 2*n =* 4*x =* 20 and *E. filiculmis* with 2*n =* 6*x =* 30, showed intermediate 2C values of 0.46 and 0.53 pg, respectively (Table 1). These data suggest that an increase in 1C DNA content follows an increase in ploidy levels, with a positive Pearson correlation (*R*^2^ = 0.712) between ploidy and C*x* values (Fig. 1A, B). Investigation with ANOVA showed that C*x* values are significantly different (*P* <0.05). The Tukey test (HSD) allowed us to separate the species according to the highest and lowest mean values (Table 1). To sum up, phylogenetically close species such as *E. maculosa*, *E. geniculata* and *E. sellowiana* (sect. *Eleogenus*) have a lower and similar C*x* value, while *E. montana* and *E. elegans* (sect. *Eleocharis*), and *E. niederleinii* (sect. *Tenuissimae*) exhibited relatively larger amounts of DNA by chromosome complement.

In general, the mean C*x* values agreed with karyotype structures. Holocentric chromosomes were observed in all the species (Supplementary Data Fig. S2), and those with similar DNA contents also have a similar chromosome number and size. We observed karyotype similarities between *E. geniculata* and *E. maculosa*, both with 2*n* = 10 (Supplementary Data Fig. S2B, C), and also between *E. geniculata* and *E. sellowiana*, both with 2*n* = 20 (Supplementary Data Fig. S2D, F). However, it is interesting to note that, except for the karyotypes of *E. maculosa* (2*n* = 6) and *E. niederleinii* with 2*n =* 42 (Supplementary Data Fig. S2A, I), the other species show similar chromosome sizes (approx. 0.1 pg per chromosome). *Eleocharis maculosa* and *E. niederleinii* showed greater karyotype asymmetry, most probably due to fission and fusion events. In the case of *E. niederleinii*, the four larger chromosomes were at least twice the size of the smaller ones, and have multivalent pairing at meiosis (data not shown). *Eleocharis montana*, with 2*n =* 40 and regular meiosis, also exhibited an asymmetric karyotype, but rearrangements were not noted (Supplementary Data Fig. S2H).

### Comparative genomic analysis

**Fig. 2.**
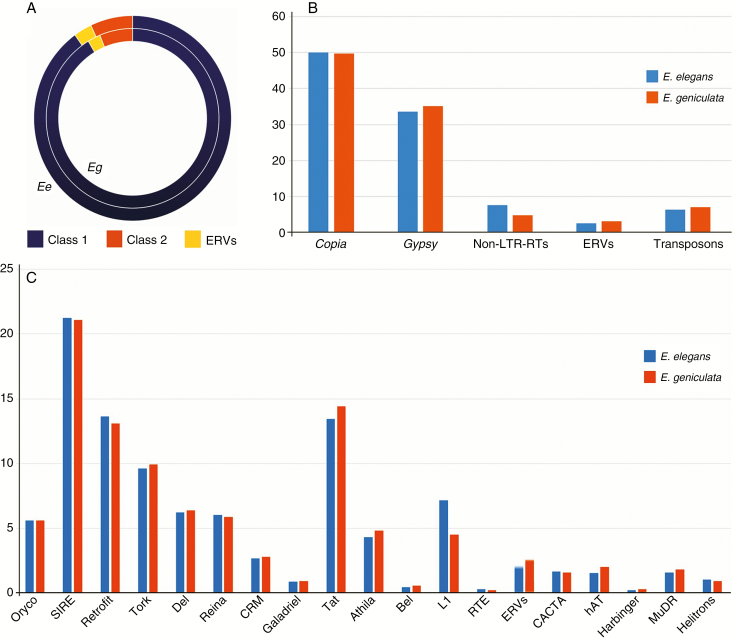
Relative distribution (%) of repetitive DNA (Classes 1 and 2) in the genomes of *E. elegans* and *E. geniculata*, based on the assembly of low coverage reads after Illumina sequencing. Note in (A) that LTR-RTs (Class 1) were more abundant in the data set than Class 2 elements and integrated viruses. (B) Relative distribution (%) of *Copia* and *Gypsy* superfamilies in the genomes of *E. elegans* and *E. geniculata*. (C) Observe that Oryco, SIRE and Retrofit of Sirevirus (*Copia*) and Athila/Tat families (*Gypsy*) are predominant in these two data sets.

A low coverage sequencing of *E. elegans* and *E. geniculata* genomes was used, which was enough for the assembly of thousands of sequences (Supplementary Data Table S1). The search for conserved protein regions of TEs in these sequences indicated homologies on approx. 10 % of each data set. Conserved proteins of Class 1 elements were the most abundant in both genomes, accounting for 91.42 % of TE homologies in *E. elegans* and 90.05 % in *E. geniculata* (Fig. 2A; Supplementary Data Table S3). Of these sequences, 83.96 and 85.31 % were LTR-RTs, respectively (Fig. 2B; Supplementary Data Table S3). Class 2 elements constituted approx. 6.81 % and approx. 6.93 % of TE homologies in *E. elegans* and *E. geniculata*, respectively (Fig. 2B; Supplementary Data Table S3). The endogenous retrovirus (ERV) family represented from 2.40 to 3.01 % of the TE homologies in *E. elegans* and *E. geniculata* data sets, respectively (Fig. 2B; Supplementary Data Table S3).

**Fig. 3.**
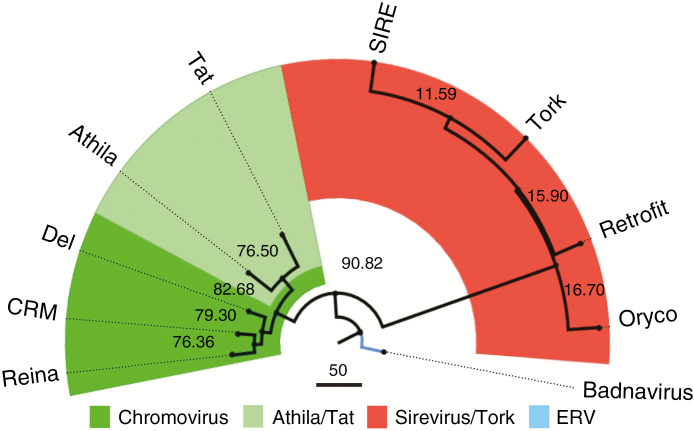
Genetic diversity graph obtained after alignment of reverse transcriptase conserved sequences with ClustalW and Muscle, elaborated with the FigTree tool. Reverse transcriptase sequences were grouped and well delimited according to the phylogeny proposed by Llorens *et al.* (2009), i.e. Sirevirus (Oryco, SIRE and Retrofit) and Tork of *Copia*, and Chromovirus (Del, Reina and CRM) and Athila/Tat of *Gypsy*

Homologies to conserved LTR-RT proteins of *Copia* and *Gypsy* superfamilies were found on 50.03 and 33.48 % of assemblies in *E. elegans*, and on 49.62 and 35.11 % in *E. geniculata*, and were within mapped sequences in both data sets (Fig. 2B). Of these, the Sirevirus and Athila/Tat clades were the most abundant (see Fig. 2C). BLASTx alignment using mapped TE sequences to detect conserved reverse transcriptase sequences (>80 % identity) allowed us to separate *Copia* elements from *Gypsy* elements (Fig. 3). However, the recognition of *Copia* families was not so accurate, different from those of *Gypsy* which were approx. 90 % similar (Fig. 3). One sequence of each identified TE family was chosen for primer design (Supplementary Data Table S2). When these primer pairs were used in PCR on the genomes of seven *Eleocharis* species, fragments corresponding to the predicted size were observed (see Fig. S3; Supplementary Data Table S2). The alignment of the sequenced PCR products showed about 60 % identity (E-value 10e-4) with their respective members in the databases (Supplementary Data Tables S4 and S5), indicating that primer design was appropriate for each clade. The data set was also used to check the GC/AT content. The GC estimates for *E. elegans* and *E. geniculata* genomes showed values of 40 and 34 %, respectively. These low GC values were also observed after genome assembly, as well as in the TE fraction (Table 2).

**Table 2.** Comparison of GC content of *E. elegans* and *E. geniculata* obtained from Illumina output files, raw reads and sequences containing TE stretches against values determined by flow cytometry

Species	TBN	Nucl. B	GC (r.r.)	GC (m.r.)	TBCensor	GC-TEs	Cov
*E. elegans*	42 644 322	42 467 196	40 %	37.3 %	464 713	31 %	2.5×
*E. geniculata*	8 312 326	8 167 100	34 %	32.3 %	194 600	23.6 %	5.5×

TBN, total number of bases of output Illumina files; Nucl. B, number of bases of raw reads, without chloroplast and mitochondrial sequences; GC (r.r.), percentage of GC of raw reads; %GC (m.r.), percentage of GC in mounted reads, without organelle sequences; TBCensor, total base number that was masked in the *Eleocharis* species using Censor/RepBase against the arabidopsis and *Oryza* genomes databases; GC-TEs, GC percentage of TEs in each genome based on RepBase alignment data; Cov, redundancy of genome coverage of sequences.

### *In situ* hybridization using LTR-RT probes

**Fig. 4.**
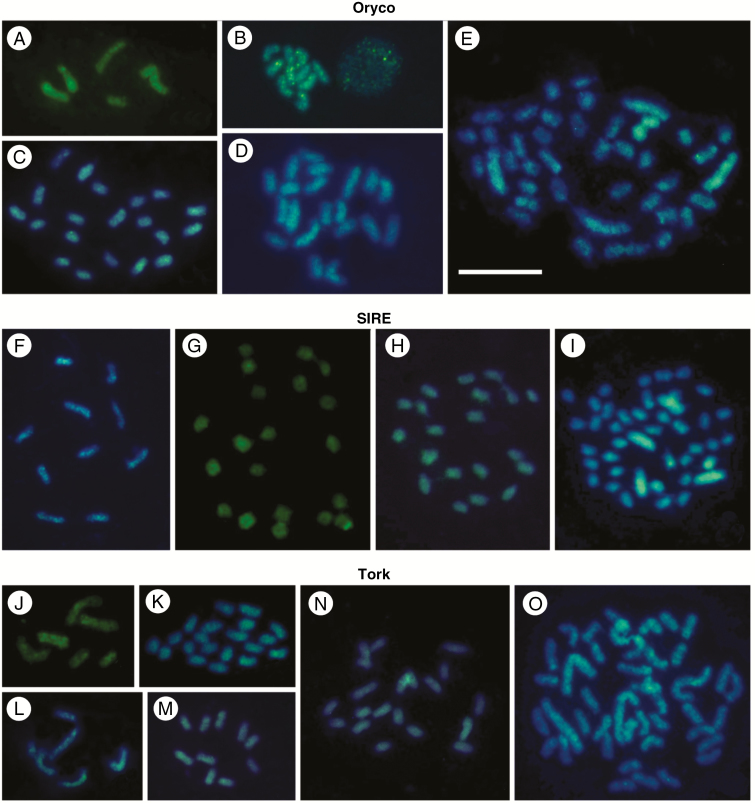
*In situ* hybridization using *Copia* LTR-RT family probes against holocentric chromosomes of diploid and polyploid *Eleocharis* species. Images appear organized according to Sirevirus (Oryco and SIRE) and Tork clades. Chromosomes were stained with DAPI (blue) and probes labelled with biotin-11-dUTP and detected with avidin–FITC conjugate (green). The Oryco probe hybridized to several small clusters in *E. maculosa* 2*n* = 6 and 2*n* = 10 (A and B, respectively). Differences were observed between *E. geniculata* (C), *E. elegans* (D) and *E. niederleinii* (E), especially in the number of hybridized chromosomes that have intense signals. In *E. niederleinii* (E), for example, the four large chromosomes (associated with dysploidy) exhibited contrasts in FISH signal accumulation. The SIRE probe showed small clustered signals in *E. maculosa* with 2*n* = 10 (F), but in *E. elegans* (G), *E. sellowiana* (H) and *E. niederleinii* (I), the FISH studies reveal a predominance of scattered signals. Note that in *E. niederleinii* the four large chromosomes showed stronger homogeneous signals than seen in the others. The FISH using the Tork probe exhibited signals predominantly scattered, with differential signal accumulation between chromosomes and karyotypes. Small clustered signals were also observed. *E. maculosa* (J, L and M), *E. geniculata* (K), *E. elegans* (N) and *E. niederleinii* (O). Scale bar = 10 μm

**Fig. 5.**
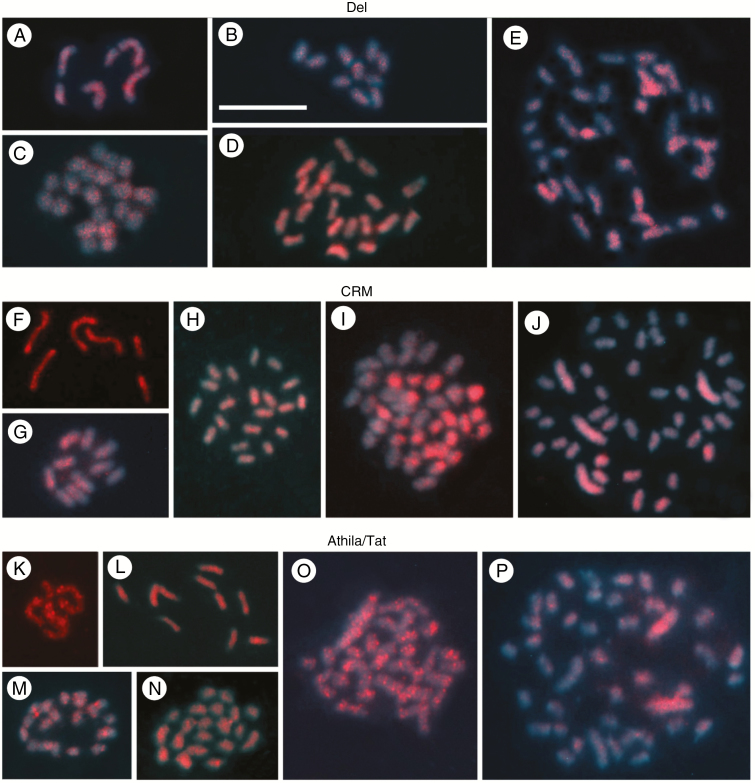
*In situ* hybridization using *Gypsy* LTR-RT family probes against holocentric chromosomes of diploid and polyploid *Eleocharis* species. Images are organized according to Chromovirus (Del and CRM) and Athila/Tat clades. Chromosomes were stained with DAPI (blue) and probes labelled with Cy3-11-dUTP (red). The Del probe hybridized in a dispersed manner along chromosomes, but in *E. maculosa* 2*n* = 6 and 2*n* = 10 (A and B) and *E. elegans* (C), signals appeared homogeneously in all chromosomes. In contrast, *E. geniculata* (D) and *E. montana* (E) exhibited stronger signals on half of the chromosomes. The CRM probe showed a slightly larger diversity in FISH location, with hybridization signals varying within chromosomes, such as in *E. maculosa* with 2*n* = 6 and 2*n* = 10, (F) and (G), respectively, as well as within the karyotypes in *E. geniculata* (H). In *E. montana* (I) and *E. niederleinii* (J), part of the chromosomes exhibited stronger FISH signals. Note also that, in *E. niederleinii* (J), the four larger chromosomes derived from dysploidy were the ones that accumulated more FISH signals with the CRM probe. The Athila/Tat probe was the one that exhibited the greatest diversity of FISH signal distribution among the *Gypsy* probes. *Eleocharis maculosa* with 2*n* = 6 and 2*n* = 10 (K and L, respectively), and *E. sellowiana* (M) exhibited scattered and clustered signals. In *E. geniculata*, with 2*n* = 20 (N), four chromosomes accumulated signals along chromatids, while the remaining FISH signals were finer and homogeneously dispersed. *Eleocharis montana* (O) exhibited numerous clustered signals, in well-delimited blocks or dots in all chromosomes. In *E. niederleinii* (P), Athila/Tat signals predominate in two of the four dysploid chromosomes, and signals with less intense brightness appeared in 16 other chromosomes with intermediate size. In the remaining chromosomes, signals were inconspicuously dispersed. Scale bar = 10 μm.

Fluorescence *in situ* hybridization assays were conducted with biotin-labelled *Copia* probes that were detected with avidin–FITC (green), and *Gypsy* probes labelled with Cy3-dUTP (red). *Copia* and *Gypsy* probes showed either scattered or clustered FISH signals, depending on the species and the LTR-RT family analysed. In some cases, probes were clustered while other species–probe combinations yielded hybridization results that were broadly scattered across the investigated genome. Figures 4 and 5, and Supplementary Data Figures S4–S9 give examples of hybridization results, with both *Copia* and *Gypsy* family probes. Differences within and between karyotypes were also found in the FISH profiles. *Copia* probes (Fig. 4) yielded predominantly scattered signals (those dispersed along chromosomes without evidence of large clusters of the same TE family), while *Gypsy* probes showed a trend toward more clustered signals (Fig. 5). Results are presented for each probe below.

Of the *Copia* TEs, the Oryco probe of Sirevirus hybridized with both scattered and small clusters on chromosomes of *E. maculosa* (Fig. 4A, B; Supplementary Data Fig. S4A–G) and *E. geniculata* (Fig. 4C; Supplementary Data Fig. S4H–J). However, in *E. maculosa*, scattered signals were seen in the sample with 2*n* = 6, while clustered signals appeared in the sample with 2*n* = 10 (Supplementary Data Fig. S4D–G). Predominantly scattered signals were observed in *E. elegans* (Fig. 4D; Supplementary Data Fig. S4K–M), *E. montana* (Supplementary Data Fig. S4N–P) and *E. niederleinii* (Fig. 4E; Supplementary Data Fig. S4Q–S) with this probe. Remarkably, the latter three species exhibited a set of chromosomes that were only weakly labelled. The SIRE probe showed a hybridization profile scattered along chromosomes, and rare clusters (Fig. 4F–I). As seen with the Oryco probe, some chromosomes accumulated more FISH signals in relation to the others, such as in *E. elegans* (Supplementary Data Fig. S5D–F), *E. sellowiana* (Supplementary Data Fig. S5G–I) and *E. niederleinii* (Fig. 4I; Supplementary Data Fig. S5M–O). The Tork and SIRE probes hybridized in a scattered fashion in all chromosomes, without evidence of large clusters (Fig. 4J–O). As seen before in some species, karyotypes were differentially labelled, with signals more evident in some chromosomes, such as in *E. geniculata* (Supplementary Data Fig. S6G–I) and *E. montana* (Supplementary Data Fig. S6M–O; see also the boxes). Interestingly, the larger chromosomes, especially those of *E. niederleinii*, exhibited a higher density of hybridization signals than smaller chromosomes.

Probes for *Gypsy* families, including members of the Chromovirus (Del and CRM) and Athila/Tat clades, exhibited more clustered signals, besides scattered signals. However, in all cases, there was great variability within and between karyotypes. The Del probe yielded a FISH profile with scattered and clustered signals, but with clear intra- and interspecific variations (Fig. 5; Supplementary Data Fig. S7). In *E. maculosa* (2*n* = 6), signals were distributed along chromosomes, with more signals in the largest chromosome (Fig. 5A; Supplementary Data Fig. S7A–C), while in *E. maculosa* (2*n* = 10) and *E. elegans* (2*n* = 20) signals were homogeneously distributed (Fig. 5B, C; Supplementary Data Fig. S7D–F, J–L). *Eleocharis geniculata* (Fig. 5D; Supplementary Data Fig. S7G–I), *E. montana* (Supplementary Data Fig. S7M–O) and *E. niederleinii* (Fig. 5E; Supplementary Data S7P–R) exhibited strong accumulations of Del homologues on almost half of the chromosomes. Similarly, the CRM probe exhibited both scattered and clustered signals, while *E. maculosa* (Fig. 5F, G; Supplementary Data Fig. S8A–D), *E. geniculata* (Fig. 5H; Supplementary Data Fig. S8I–K) and *E. elegans* (Supplementary Data Fig. S8L–N) karyotypes yielded particularly strong CRM FISH signals on almost half of all chromosomes. In *E. montana* (Fig. 5I; Supplementary Data Fig. S8O–Q) and *E. niederleinii* (Fig. 5J; Supplementary Data Fig. S8R-T) strong CRM FISH signals were observed on only a few chromosomes.

Probes of the *Gypsy* Athila/Tat families, in contrast to the probes of other LTR-RT families, exhibited FISH signals predominantly distributed in clusters, such as in *E. maculosa* with 2*n* = 6 (Fig. 5K; Supplementary Data Fig. S9A–D). In *E. maculosa* with 2*n* = 10 (Fig. 5L; Supplementary Data Fig. S9E–G), *E. geniculata* (Fig. 5N; Supplementary Data Fig. S9H–J) and *E. sellowiana* (Fig. 5M; Supplementary Data Fig. S9L–N), FISH signals for Athila/Tat appeared scattered along chromosomes. *Eleocharis elegans*, on the other hand, showed predominantly small clustered signals on the chromosomes (Supplementary Data Fig. S9O–Q). The Athila/Tat probe exhibited a greater amount of clustered signals in *E. montana* (Fig. 5O; Supplementary Data Fig. S9R–T), but this probe in *E. nierderleinii* showed weak and predominantly scattered signals, except for two large chromosomes that have accumulated signals along almost all of their length (Fig. 5P; Supplementary Data Fig. S9U–X). In this case, it is important to mention that this probe hybridized to only two of the four large chromosomes, while CRM and Del probes hybridized to all four.

## DISCUSSION

### C-value and its influence on karyotype differentiation

The amount of DNA in plants varies >2000-fold, from approx. 63 Mbp in *Genlisea aurea* to approx. 14 900 Mbp in *Paris japonica* (Bennett and Leitch, 2011). Polyploidization events, whether auto- or allopolyploidy, as well as the accumulation and elimination of repetitive DNA families and differential activity of TEs, are the main factors responsible for DNA content fluctuations (Kellogg and Bennetzen, 2004; Grover and Wendel, 2010; Bureš and Zedek, 2014). Since holocentric species seem to tolerate chromosome rearrangements, such as fission or fusion events (Luceño and Castroviejo, 1991; Da Silva *et al.*, 2008), a combined analysis of chromosome counting and DNA content estimation is required to determine the relationship between chromosome number change and DNA content flexibility (see Bureš *et al.*, 2013). Data obtained here for South American *Eleocharis* (subgenus *Eleocharis*) demonstrated that polyploidy is a primary evolutionary mechanism for DNA content amplification, and it is in agreement with the strong positive correlation between the amount of DNA and polyploidy observed in *Eleocharis* species from Europe (Zedek *et al.*, 2010). However, these same authors suggested that in the subgenera *Limnochloa* and *Zinserlingia*, that exhibit karyotypes with numerous and small chromosomes, fission (agmatoploidy) may have played an additional role in DNA C-value variation.

Fluctuations in the amount of DNA are common during eukaryotic genome evolution, and they can be motivated by large- or small-scale rearrangements, such as polyploidy and dysploidy. In this sense, *E. niederleinii* is an interesting example because it presents 2*n* = 42 (C*x* = 0.63 pg) and four large chromosomes derived from fissions and fusions (see Da Silva *et al.*, 2017). Chromosomes of *E. niederleinii* exhibited an accumulation of AT-rich terminal heterochromatin, and the four large chromosomes showed the greatest accumulation of LTR-RT FISH signals, compared with the others. This evidence may explain the increase in C*x* value in relation to the other studied species. Intraspecific variation in polyploids was detected in *E. geniculata* with 2*n* = 10 (C*x* = 0.50 pg) and 2*n* = 20 (C*x* = 0.46 pg), as well as in the dysploid *E. maculosa* with 2*n* = 6 (C*x* = 0.43 pg) that exhibited a decrease in C*x* value in relation to samples with 2*n* = 10 and C*x* = 0.49 pg. As an example of interspecific variation in DNA content, we can mention the approx. 30 % difference between *E. sellowiana* C*x =* 0.41 pg (2C = 1.64 pg) and *E. elegans* C*x =* 0.58 pg (2C = 2.34 pg), both with 2*n* = 20. Although it is clear that the small-scale variations for DNA C-value in *Eleocharis* may involve changes in the repetitive fraction and dysploid changes, this does not seem to be a rule for sedges. In *Carex*, for instance, the increase in chromosome number due to rearrangements does not involve large fluctuations in the amount of DNA (Roalson *et al.*, 2008; Chung *et al.*, 2011, 2012). In *Luzula* (Juncaceae), another group with holocentric chromosomes, Bozek *et al.* (2012) did not find a clear correlation between chromosome number and genome sizes. The fact is that DNA C-value changes in plants may be a result of multifactorial and simultaneous events, involving numerical and structural rearrangements and activity of TEs (Grover and Wendel, 2010; Bennetzen and Wang, 2014). Good examples of the influence of LTR-RT activity on genome size were the differential amplifications of the *Copia* element BARE-1 in *Hordeum* (Vicient *et al.*, 1999), and the *Gypsy* element Ogre in *Vicia* (Neumann *et al.*, 2006). It is believed that the increase in plant genome sizes due to an increase in LTR-RT content could consequently lead to an increase in GC content (Šmarda *et al.*, 2014). However, this contrasts with the low GC amounts found in the genomes of *E. elegans* and *E. geniculata*. According to Šmarda and Bureš (2012) and Šmarda *et al.* (2014), holocentrics can exhibit reduced recombination and repair rates, which could maintain low GC levels in comparison with plants with monocentric chromosomes. Although these data could explain the low GC amounts found in two genomes of *Eleocharis*, additional data from flow cytometry and molecular analysis in other species are required to confirm this trend.

Transposable elements, especially LTR-RTs, can also cause an ‘elastic effect’ in the DNA C-value, due to differential events of amplification and removal of each TE family in different chromosomes or chromosome regions (Heslop-Harrison and Schwarzacher, 2011; Tenaillon *et al.*, 2011; Santos *et al.*, 2015). An expectation could be created in relation to the possible tolerance of holocentric chromosomes to rearrangements (Zedek and Bureš, 2018), especially on the role of LTR-RTs in rearranged karyotypes, such as those with even and odd numbers, irregular meiosis or with high asymmetry, which is frequently found in Cyperaceae (Da Silva *et al.*, 2008, 2017; Roalson, 2008). It is proposed that karyotype changes could constitute a kind of genomic stress, acting in favour of differential activity of TEs (Bureš and Zedek, 2014). Although new experiments focused on these events are necessary to elucidate this issue, this could be one explanation for the observed differences in DNA content and in the fluctuation of *Copia* and *Gypsy* families between *E. elegans*, *E. sellowiana* and *E. geniculata*.

### Comparative cytogenomic analysis

Recent advances in DNA sequencing technologies, bioinformatics tools and databases with more reliable annotations have provided the resources for the scientific community to broaden its understanding of the genomic composition of native species. Although it was possible for us to compare two partially sequenced genomes of *E. elegans* and *E. geniculata* to produce a TE portfolio, our effort was directed to identify and investigate mainly the sequences related to the conserved protein regions in the TEs. It is widely known that retroelements represent a large portion of monocotyledonous genomes, and LTR-RTs account for an important portion of the TE DNA in plants (Bennetzen and Wang, 2014). In maize, for instance, the percentage of LTR-RT DNA reaches approx. 75 % of the total nuclear genome (Schnable *et al.*, 2009; Devos, 2010). The annotated TEs from data sets of *E. elegans* and *E. geniculata* indicate a trend toward LTR-RTs accumulation. However, we do not consider this underprediction of their genome contribution, because the low coverage does not allow a perfect assembly of complete elements, and because the TE identification in native species, without good references, could lead to misidentification of many TEs.

In general terms, approx. 50 % of the LTR-RTs identified in *Eleocharis* were *Copia* and approx. 35 % were *Gypsy*. Other plant genomes have presented more pronounced differences in the superfamily abundance, such as in *Musa* with 25.7 % *Copia* and 11.6 % *Gypsy* (D’Hont *et al.*, 2012) and *Helianthus* and *Solanum*, with a high predominance of *Gypsy* members (Paz *et al.*, 2017; Qiu and ungerer, 2018). In *Brachiaria* and *Festuca*, both Poaceae, *Gypsy* are also most abundant (Santos *et al.*, 2015; Křivánková *et al.*, 2017). In the holocentric species *Luzula elegans* (Juncaceae), it was found that approx. 33 % of its genome is composed of *Copia*, with only approx. 1.1 % belonging to *Gypsy* elements (Heckmann *et al.*, 2013), and in *Rhynchospora pubera* (Cyperaceae), *Copia* members seem to be more abundant than *Gypsy* elements (Marques *et al.*, 2015).

From the point of view of karyotypes and chromosomal biology, genomic understanding restricted to *Gypsy* and *Copia* superfamilies is somewhat superficial, because the diversity of the repetitive DNA fraction depends not only on the evaluated genomes but also on the evolutionary history, positioning and role of each repetitive sequence type [simple sequence repeats (SSRs), satDNA, TEs and others] in the genomes (Heslop-Harrison and Schwarzacher, 2011). Based on TE phylogeny (Llorens *et al.*, 2009), the relative differences in the occurrence of *Copia* and *Gypsy* elements when comparing the genomes of *E. elegans* and *E geniculata* indicates a predominance of sequences from the Sirevirus and Athila/Tat clades, and low occurrence of the Chromovirus clade. In addition, the variation among these families in *Eleocharis* suggests that, although all LTR-RTs have a similar transposition mechanism, each LTR-RT family exhibits an independent activity history and differences in the relative quantity of autonomous and non-autonomous elements, and these facts may determine their genomic distribution profile, as suggested by Bennetzen and Wang (2014).

As previously mentioned, low coverage sequencing makes it difficult to obtain large sequences, and it hinders the reconstruction of complete LTR-RTs. However, short sequence alignment (from approx. 200 to 800 bp length) against databases that contain highly conserved protein domains, especially for the POL region of the LTR-RTs, allows a secure selection of useful sequences to recognize clades and families of TEs (Langdon *et al.*, 2000; Dixit *et al.*, 2006), as well as for FISH probe production. Probes of highly conserved sequences from POL of *Copia* (Oryco, SIRE, Retrofit and Tork) and *Gypsy* (Del, CRM, Athila and Tat) allow detection of differences in frequency and chromosomal location of each family (see Santos *et al.*, 2015), and this strategy was successfully applied to seven *Eleocharis* species. In general, chromosomes of *Eleocharis* showed *Copia* probes with a more scattered signal distribution after FISH, whereas *Gypsy* signals were scattered using the Del probe and more clustered with the Athila/Tat and CRM probes. This variability is especially dramatic in *E. montana*, where Oryco, Del and CRM probes hybridized to half of the chromosomes in this species, in contrast to what happened with the Athila/Tat probe, where clustered signals were evident on all chromosomes. The Athila/Tat probe, for example, appeared in a scattered manner in *E. nierderleinii*, especially in the two larger chromosomes that are originated by symploidy (for details of chromosome fusion and fission, see Da Silva *et al.*, 2017).

The literature indicates that retroelements generally accumulate in scattered patterns across plant chromosomes, unlike satellite DNAs that form more defined clusters (Heslop-Harrison and Schmidt, 2007; Heslop-Harrison and Schwarzacher, 2011; Ribeiro *et al.*, 2016). However, there are examples that report that *Copia* and *Gypsy* members have a heterogeneous profile, being able to be located either as scattered TEs, clustered TEs or a combination of both on some chromosomes (Belyayev *et al.*, 2001; Gaeta *et al.*, 2010), including holocentric chromosomes (Heckmann *et al.*, 2013; Marques *et al.*, 2015). FISH using *Copia* and *Gypsy* families in these *Eleocharis* species increases our knowledge of the location and distribution of LTR-RTs in holocentric genomes. In the single previous holocentric example, for *Luzula elegans*, *Copia* and *Gypsy* probes that were not specific to any LTR-RT family showed scattered FISH signals, while *Gypsy* also exhibited slightly clustered signals (Heckmann *et al.*, 2013). Our data lead us to some questions. (1) Why are some LTR-RTs found more abundantly in some *Eleocharis* species and chromosomes than in others? (2) Why are some clustered and some not clustered? Probably, this difference is caused by such factors as the potential activity of each family in each genome, epigenetic controls in different genomes or chromosomal regions, the influence of neighbouring sequences of other natures, as well as an association with chromosomal rearrangements. The possible involvement of these factors needs to be studied further.

Our FISH results reinforce the idea that different families of LTR-RTs have independent activity on genomes and chromosomes, with different evolutionary histories and fates. As an example of this postulate, we can mention the differences in the CRM family probe distribution pattern. CRM, first named as a centromeric retrotransposon of maize, was detected in the centromere-proximal chromosome regions of several species (Neumann *et al.*, 2011; Nunes *et al.*, 2018), and associated with the holocentromeres of *Rhynchospora* (Marques *et al.*, 2015; Rocha *et al.*, 2016). Members of the CRM family have a terminal chromodomain in the polygenic transcript, with a zinc finger domain (HHCC) that recognizes the CENH3 protein. This chromodomain may direct its accumulation preferentially into or near centromeres, which may differentiate it from other LTR-RT families that lack this specific chromodomain (Houben *et al.*, 2007; Neumann *et al.*, 2011). Some chromosomes of *E. maculosa*, *E. elegans* and *E. montana* showed FISH signals distributed along the chromatids, agreeing with the holocentric condition. However, other chromosomes did not exhibit continuous signals using the CRM FISH probe, but rather scattered signals or as very small clusters. In addition, in species such as *E. montana* and *E. niederleiini*, some chromosomes exhibited strong signals with the CRM probe, but others did not. The CRM probe used against the *Eleocharis* chromosomes was designed for the RNase H fraction of this family, regardless of the presence or absence of a chromodomain or CR motif, i.e. this probe was generic to the CRM clade. This could explain why the signals were also scattered and not specifically located in the holocentromeres. The holocentromeric organization observed in *Rhynchospora* (Marques *et al.*, 2015) apparently does not occur in *Eleocharis*.

Studies on the role of LTR-RTs in the evolution of genomes and karyotype in holocentric species are still scarce when compared with organisms with monocentric chromosomes. This lack of information makes it difficult to establish evolutionary models for organisms with a large incidence of chromosomal rearrangements, such as the dysploidy commonly found in holocentrics of Cyperaceae. Nonetheless, this study presents conclusive evidence that rearranged chromosomes accumulated more LTR-RTs when compared with the others, as observed in *E. nierderleinii*. Likewise, our data showed that the polyploids with the highest C*x* values have a higher density of FISH signals per chromosome than the diploids, for example in the comparison between *E. montana* and *E. maculosa*. This leads us to believe that the differential LTR-RT activity has played a secondary role in *Eleocharis* karyotype diversity when compared with ploidy variation.

This comparative analysis between TE activity and numerical chromosomal rearrangements is especially interesting in this case, because we compared holocentric species of *Eleocharis* with different karyotype conditions: diploid, polyploid and dysploid. Even though it is obvious that TE activity and polyploidy are the major sources of plant genomic variations (see Bennetzen, 2005; Vicient and Casacuberta, 2017), the current study showed differences in LTR-RT family fates and their contributions to DNA C-value fluctuations, associated with contrasting genomic histories. An example was the greater accumulation of FISH signals with the Athila/Tat and CRM probes of *Gypsy* in relation to the *Copia* signals, in different chromosomes and species, mainly in those chromosomes which are known to be rearranged. Hence, rapid chromosome change involving LTR-RTs seems to be an important mechanism responsible for genome differentiation in *Eleocharis*, subgenus *Eleocharis*. This contribution opens up new perspectives for studying species of other subgenera of *Eleocharis*, as well as species of other genera that are known to have karyotypes with numerous and small chromosomes, such as *Cyperus*, *Carex* and others, and this will help us improve our understanding of the processes involved in karyotype differentiation in Cyperaceae.

## SUPPLEMENTARY DATA

Supplementary data are available online at https://academic.oup.com/aob and consist of the following. Table S1: information on genomic sequences. Table S2: information on the primers and LTR-RT families. Table S3: distribution of TEs in the genomes. Table S4: consensus sequences of the PCR products that were used as probes. Table S5: alignment of the PCR product sequences. Figure S1: data from flow cytometry. Figure S2: mitotic chromosomes Giemsa stained. Figure S3: electrophoresis gel with PCR products of the POL chain stretches. Figures S4–S9: FISH using retrotransposon probes separately for *Copia* and *Gypsy* families.

Supplementary Data
