# Supplementary material for: Analysis of retrotransposon abundance, diversity and distribution in holocentric Eleocharis (Cyperaceae) genomes
Source: Ann Bot. 2018 May 4;122(2):279–90. doi: 10.1093/aob/mcy066 (PMC6070107; doi:10.1093/aob/mcy066)
Supplement: Supplementary Data [file mcy066_suppl_supplementary-data.docx]

**Supplementary data**

Analysis of retrotransposon abundance, diversity and distribution in holocentric *Eleocharis* (Cyperaceae) genomes.

Thaíssa B. Souza^1^, Srinivasa R. Chaluvadi^2^, Lucas Johnen^1^, André Marques^3^, M. Socorro González-Elizondo^4^, Jeffrey L. Bennetzen^2^, and André L. L. Vanzela^1*^

**Supplementary Tables S1- S5 and Supplementary Figures S1- S9**

**Table S1**. Information on assembled contiguous sequences from *E. elegans* and *E. geniculata* genomes, using Illumina sequencing and the SPAdes assembler tool.

| **Species** | **Input reads** | **K-mers** | **Sequence numbers** | **Largest sequence** | **TE sequences** |
| --- | --- | --- | --- | --- | --- |
| *E. elegans* | 5,448,113 | 31, 51, 91 | 142,822 | 15,808 | 11,758 |
| *E. geniculata* | 1,177,302 | 51, 61, 71 | 27,185 | 43,190 | 4,819 |

Input reads = number of sequence reads in the Illumina Miseq PE250 output files obtained from FastQC. K-mers = odd K-mer values used in the SPAdes assembly process. Sequence numbers = total number of sequences per genome. Largest sequence = length of longest sequence (bp). TE sequences = number of sequences containing stretches of identified TEs.

**Table S2.** Information on the primers designed for FISH and PCR amplification of TE fragments.

| **Lineages** | **Primers** | **FS (bp)** | **Protein domain** | **Sequences** |
| --- | --- | --- | --- | --- |
| Oryco | F GCCGGGCCATTAGTATTTTC | 410 | INT | EG118 |
|  | R CGGCTCCTAGAACACCACA |  |  |  |
| SIRE | F CACTTGCCGAAGGTTTCTCT | 350 | RT | EG232 |
|  | R GAGACTCTTTAGGGTTCGCTTG |  |  |  |
| Tork | F CGATCCCATGAATCTGACTG | 200 | RT | EG1616 |
|  | R GACTCCAAGGCATGGCAAG |  |  |  |
| Del | F GCTCAAGAGGCACTTCTGGT | 250 | INT | EE4221 |
|  | R ACGGCGTTGAAGTTCTTCTG |  |  |  |
| CRM | F GAAGTAGAGTGCGACGCTTCA | 250 | RNaseH | EE1989 |
|  | R GTCGGCACTCAGCTTGT |  |  |  |
| Athila | F TGGTAGCGGTGGATTATGTG | 440 | INT | EE338 |
|  | R GCCCAATATGCCTTATGCTC |  |  |  |
| Tat | F CCTCCCAGAAACCTGAAACA | 200 | RT | EE2869 |
|  | R AGCCAAAACTGGTGGTTTGA |  |  |  |

FS = fragment size obtained after PCR for probe used in FISH experiments (all amplification products were of the approximate predicted size); Protein domain = conserved stretch of integrase (INT), reverse transcriptase (RT) or RNaseH that was PCR amplified; Scaffolds used for primers design for EG (*E. geniculata*) and EE (*E. elegans*).

**
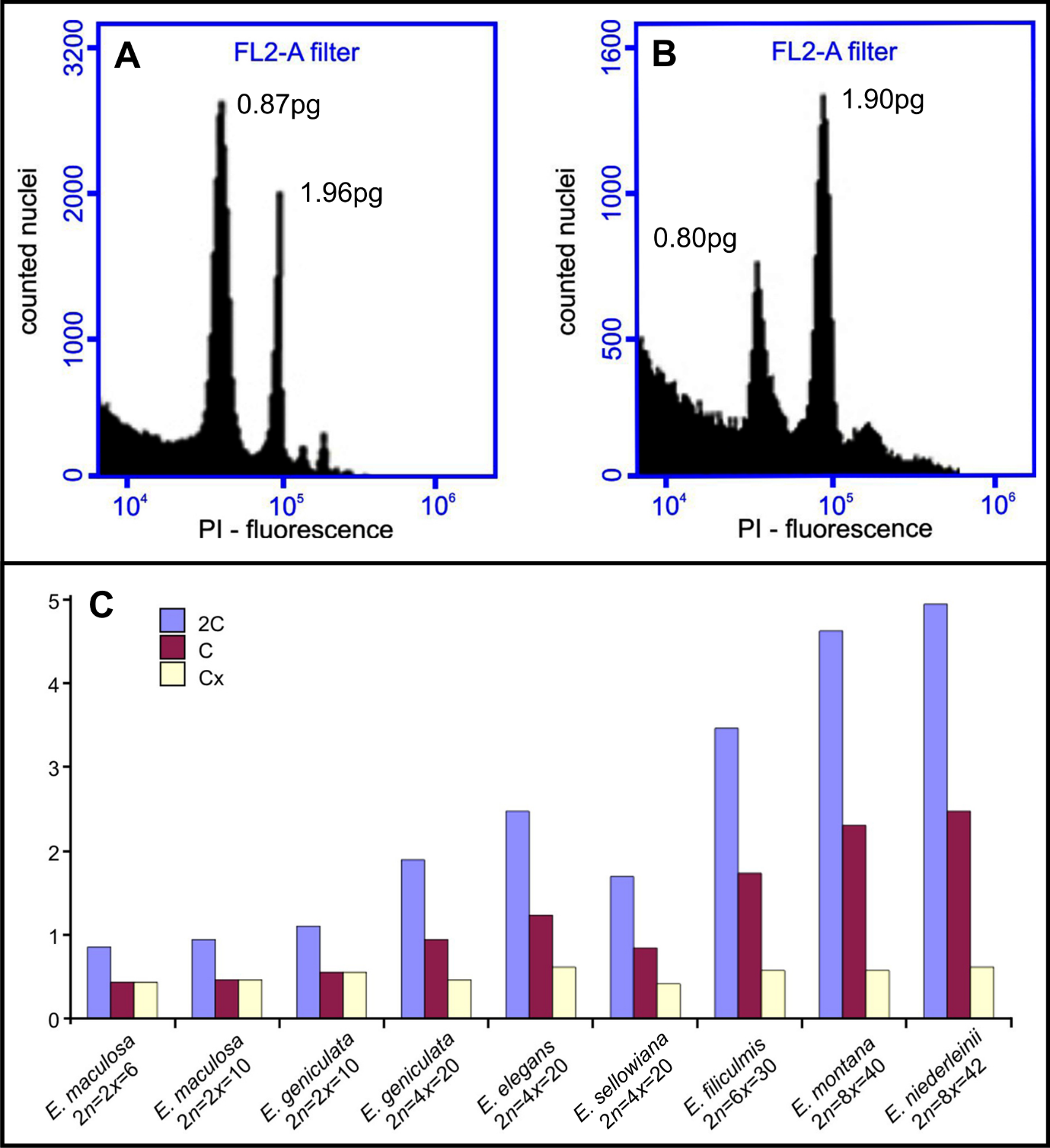
**

**Figure S1.** DNA C-value comparisons between *Eleocharis* species (*E. maculosa* with 2*n*=6 and 2*n*=10, *E. geniculata* with 2*n*=10 and 2*n*=20, *E. elegans* and *E. sellowiana*, both with 2*n*=20, *E. filiculmis* with 2*n*=30, *E. montana* with 2*n*=40 and *E. niederleinii* with 2*n*=42). (**A-B**) Histograms showing DNA amount in *E. maculosa* using *Solanum lycopersicum* (2C = 9.73 × 10^4^ fluorescence) as standard. The left peak with 0.87 pg (4.37 × 10^4^ fluorescence) represents the 2C value in *E. maculosa* (2*n*=6), while the right peak at 1.96 pg represents *S. lycopersicum* 2C value (**A**). The 1.90 pg in B (9.0 × 10^4^ fluorescence) represents *E. geniculata*, compared with the *Rhynchospora breviuscula* standard with 2C=0.80 pg and fluorescence of 3.78 × 10^4^. (**C**) Total DNA C-values, including the probable monoploid complement amount (C*x*) in picograms. Note that the increase in DNA amount was accompanied increase in ploidy, except in *E. sellowiana*, whose values were lower than expected.


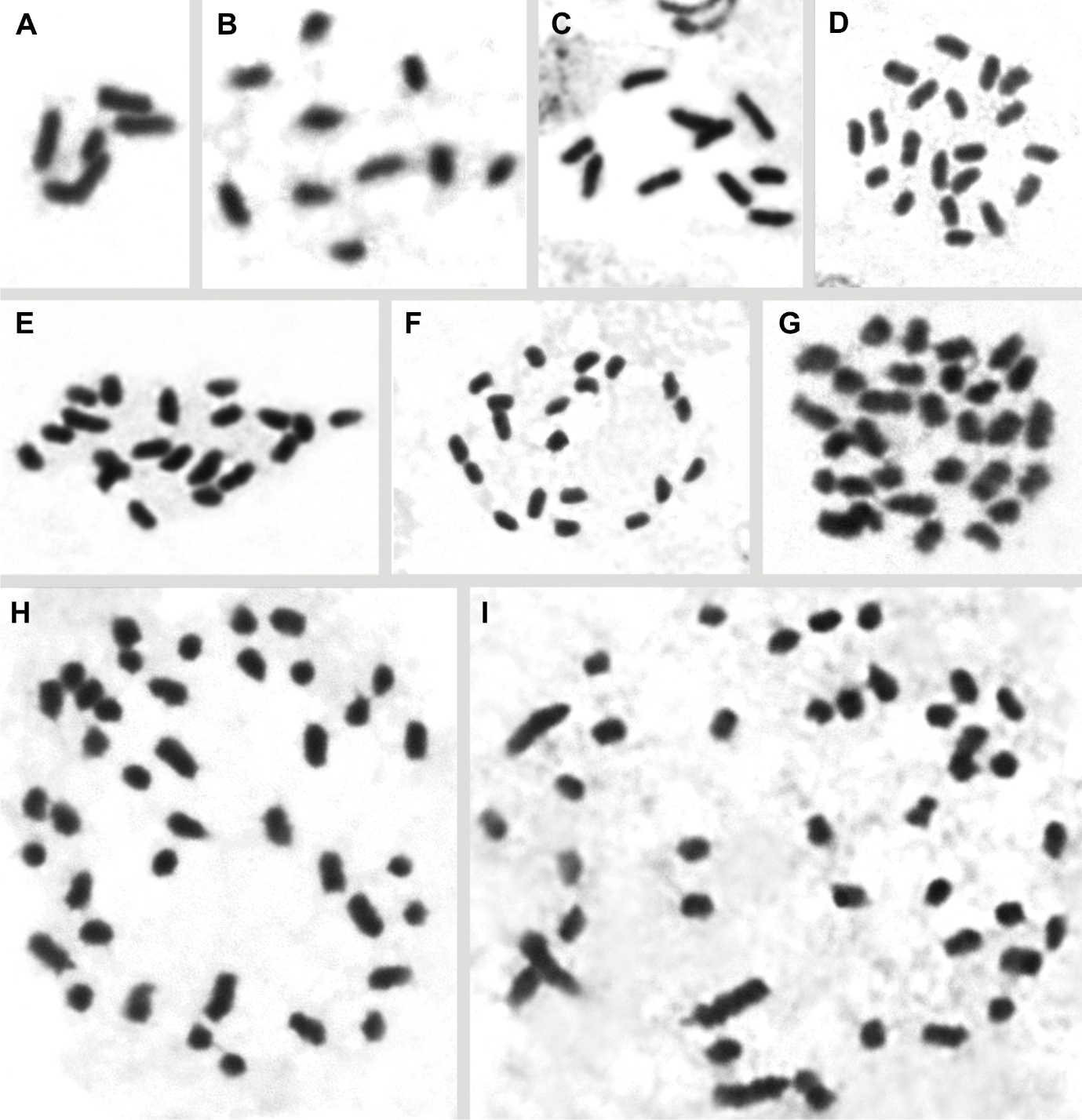


**Figure S2.** Giemsa stained mitotic chromosomes of *Eleocharis* species. (**A**) Prometaphase in *E. maculosa* with 2*n*=6. Note different sized chromosomes resulting from dysploidy. (**B-C**) Metaphases of *E. maculosa* and *E. geniculata*, both with 2*n*=10. (**D-F**) Metaphases with 2*n*=20 in *E. geniculata* (**D**), *E. elegans* (**E**) and *E. sellowiana* (**F**). Symmetrical karyotypes were observed in *E. sellowiana* and the others. (**G**) Metaphase of *E. filiculmis* with 2*n*=30, and slightly asymmetrical karyotype. (**H-I**) Metaphases in *E. montana* with 2*n*=40 and *E. niederleinii* with 2*n*=42, respectively. Note the asymmetrical karyotypes, with large chromosomes much larger than the small ones of the complement. Bar = 10 µm.

**Table S3.** Distribution of TEs in the genomes of *E. elegans* and *E. geniculata* after assembly using the SPAdes tool.

| **Species/Lineages** | | | ***E. elegans*** | | | | ***E. geniculata*** | | | |
| --- | --- | --- | --- | --- | --- | --- | --- | --- | --- | --- |
|  |  |  | **AN** | **RN (%)** | **Amount (bp)** | **RA (%)** | **AN** | **RN (%)** | **Amount (bp)** | **RA (%)** |
| *Copia* | Oryco | | 42,715 | 5.64 | 3,240,328 | 5.58 | 10,508 | 5.39 | 706,178 | 5.59 |
|  | SIRE | | 156,770 | 20.71 | 12,325,884 | 21.22 | 39,003 | 20.00 | 2,662,194 | 21.06 |
|  | Retrofit | | 99,479 | 13.14 | 7,911,393 | 13.62 | 24,016 | 12.31 | 1,651,067 | 13.06 |
|  | Tork | | 69,255 | 9,15 | 5,587,157 | 9.62 | 17,556 | 9.00 | 1,253,122 | 9.91 |
| Total *Copia* | | | 368,219 | 48.64 | 29,064,762 | 50.03 | 91,083 | 46.70 | 6,272,561 | 49.62 |
| *Gypsy* | Del | | 44,001 | 5.81 | 3,599,784 | 6.20 | 11,338 | 5.81 | 804,620 | 6.37 |
|  | Reina | | 41,571 | 5.49 | 3,493,143 | 6.01 | 10,774 | 5.52 | 739,756 | 5.85 |
|  | CRM | | 19,310 | 2.55 | 1,555,585 | 2.68 | 5,199 | 2.67 | 353,129 | 2.79 |
|  | Galadriel | | 5,801 | 0.77 | 503,202 | 0.87 | 1,571 | 0.81 | 114,628 | 0.91 |
|  | Tat | | 91,636 | 12.10 | 7,790,839 | 13.41 | 25,144 | 12.89 | 1,817,720 | 14.38 |
|  | Athila | | 29,568 | 3.91 | 2,509,894 | 4.32 | 8,210 | 4.21 | 607,761 | 4.81 |
| Total *Gypsy* | | | 231,887 | 30.63 | 19,452,447 | 33.48 | 62,236 | 31.91 | 4,437,614 | 35.11 |
| Bel | |  | 7,286 | 0.96 | 260,893 | 0.45 | 2,274 | 1.17 | 72,960 | 0.58 |
| Total LTR-RTs | | | 607,392 | 80.23 | 48,778,102 | 83.96 | 155,593 | 79.78 | 10,783,135 | 85.31 |
| LINE | L1 | | 62,293 | 8.23 | 4,161,448 | 7.16 | 11,614 | 5.96 | 569,411 | 4.50 |
|  | RTE | | 2,808 | 0.37 | 167,319 | 0.29 | 541 | 0.28 | 27,730 | 0.22 |
| SINE |  | | 318 | 0.04 | 7,767 | 0.01 | 95 | 0.05 | 2,477 | 0.02 |
| Total Non-LTR-RTs | | | 65,419 | 8.64 | 4,336,534 | 7.46 | 12,250 | 6.28 | 599,618 | 4.74 |
| Total Class 1 | | | 672,811 | 88.87 | 53,114,636 | 91.42 | 167,843 | 86.06 | 11,382,753 | 90.05 |
| ERVs | Caulimovirus | | 6,876 | 0.91 | 427,789 | 0.74 | 2,016 | 1.03 | 111,370 | 0.88 |
|  | Soymovirus | | 3,093 | 0.41 | 173,477 | 0.30 | 882 | 0.45 | 45,596 | 0.36 |
|  | Cavemovirus | | 816 | 0.11 | 49,754 | 0.09 | 241 | 0.12 | 14,327 | 0.11 |
|  | Tungrovirus | | 30 | 0.00 | 976 | 0.00 | 13 | 0.01 | 366 | 0.00 |
|  | Badnavirus | | 11,885 | 1.57 | 744,140 | 1.28 | 3,280 | 1.68 | 208,336 | 1.65 |
| Total ERVs | | | 22,700 | 3.00 | 1,396,136 | 2.40 | 6,432 | 3.30 | 379,995 | 3.01 |
| Transposons | Cacta | | 13,661 | 1.80 | 950,867 | 1.64 | 4,152 | 2.13 | 196,795 | 1.56 |
|  | hAT | | 13,840 | 1.83 | 889,496 | 1.53 | 5,063 | 2.60 | 254,327 | 2.01 |
|  | PIF-Harbinger | | 3,848 | 0.51 | 120,496 | 0.21 | 1,467 | 0.75 | 37,995 | 0.30 |
|  | Mariner | | 1,061 | 0,14 | 29,179 | 0.05 | 390 | 0.20 | 10,103 | 0.08 |
|  | MuDR | | 14,785 | 1.95 | 916,041 | 1.58 | 5,125 | 2.63 | 230,962 | 1.83 |
|  | MITEs | | 2,377 | 0.31 | 60,262 | 0.10 | 917 | 0.47 | 22,211 | 0.18 |
| Crypton |  | | 601 | 0.08 | 16,397 | 0.03 | 222 | 0.11 | 5,329 | 0.04 |
| Helitrons |  | | 10,981 | 1.45 | 591,244 | 1.02 | 3,281 | 1.68 | 113,841 | 0.90 |
| Polintons |  | | 367 | 0.05 | 14,571 | 0.03 | 135 | 0.07 | 4,143 | 0.03 |
| Total Class 2 | | | 61,521 | 8.13 | 3,588,553 | 6.18 | 20,752 | 10.64 | 875,706 | 6.93 |

AN = absolute number of sequences; RN (%) = relative number of sequences; Amount (bp) = sum of the fragment sizes in base pairs; RA (%) = relative amounts of the added fragments.


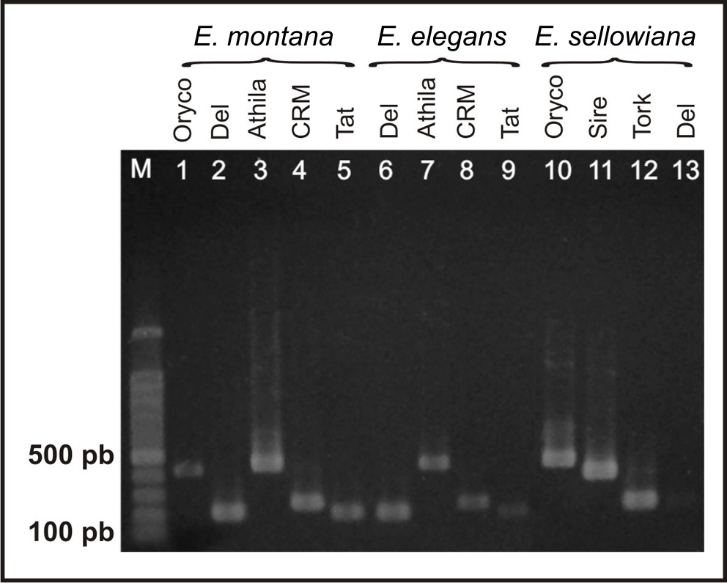


**Figure S3.** PCR products for LTR-RT POL-sequences. Lane M represents 100 bp length ladder. Lanes from 1 to 5 with primers for Oryco, Del, Athila, CRM and Tat using genome of *E. montana* as template. Lanes from 6 to 9 with primers for Del, Athila, CRM and Tat, for the *E. elegans* genome. Lanes from 10 to 13 with primers for Oryco, SIRE, Tork and Del, using the *E. sellowiana* genome.

**Table S4.** Consensus sequences of the PCR products used as probes for FISH experiments. * corresponds to alignment obtained from NCBI database. Number after NCBI corresponds to the sequence registration number at NCBI.

| **>Oryco_INT_NCBI: MG865448**  TTGGCCGGSCCATTAGTATTTTCTTTGAATYTCACATGAATAGACTCMATAATTGTTCTAGAGGTGGGAATGAAAATTCTATATGCTTTGCTAGTCAATGAGTACCCCACRAATATGCCTTCAATGCACTTAGAATCAAACTTGGTTGTCTTAGGTGACTCATCAATAACAAAGCACTTTGATCCGAATGCTCTAAAATAGGATATGTTAGGTTTTCGCCCCATCCATAGCTCATATGGTGTTTTGAGTARAGTAGGTCTAAGGCTTACTCGGTTAATTACGTAACATGCCGTATTAACCGCCTCCGCCCATAAATATGTTGGAAGAGATRACTCATTGAGCATGGTTCGGGCTAATTCTTGYAAAGTTCTATTTTTCCTTTCAACGACCCCATTTTGTTGTGGTGTTCTAGGAGCCGAAATAAAATG  * 64% identity (E-value = 2e-54) with POL_Copia_TNT-1 of *C. cajan* (KYP35263.1) |
| --- |
| **>SIRE_RT_NCBI: MG865442**  GGCACTTGCCGAAGGTTTCTCTTCTTTGATGAGTAGTGAATTTGAGATGAGCATGATGGGCGAACTCACCTTTTTCTTGGGACTTCAAATCAAACAAACTCCGGAAGGCACATTCATAAATCAAGCGAAATATGCAAAGGAGTTGATTAAGAAKTTTGGKGTAGAAGATAKYWAGAWGAGNNCAACANTCCRATGGNTRYYAWCRTCWWYMTKGACGCGGATGAAAGCGGCACACCGGTGGACATCTCCCAATACCGAGCTATGATAGGTAGTCTACTCTATTTAACGGCTAGTCGGTCGAATATTATGTTTTCAGTGTGTCTTTGCACTAGGTTT  * 65% identity (E-value = 9e-21) with POL_Copia of *O. sativa* (AAW57784.1) |
| **>Tork_RT_NCBI: MG865443**  TTCATCATAGTCGATCCCATGAATCTGACTGAAACCTTTCGCTACAAGTCTCGCCTTGTAGGTTGAYATGTTTCCATTCAAATCCATCTTCCTTTTGAAAATCCATTTGCACTGAATGGRTCTCTTTTCATCAGGAAGGTCTACCAAAGTCCAGACTTTATTATCGTACATGGACWGTATTTCGGATTCCATGGCTCCTTGCCANGCCTTGGAGTCATCTCTCTGTATAGCTTCTTCATATG  * 63% identity (E-value = 1e-25) with POL_Copia of *O. sativa* (ABG22008.1) |
| **>Del_INT_NCBI: MG865444**  CAGWSGCTCAAGAGGCACTTCTGGTGGARGYGGATGCATCGYGAGGTGGCSCAGTACGTGGCTCGATGCCTYGTATGCCAGAAGGTGAAGGCAGARCGACAGAGGCCCGCKGGACTGTTGCGGCCTCTACCGAAGTCYCAGCGCAAGTTCGACATCATTACGATGGACTTCGTCACGGGGTTGCCGAAGACGCAGAAGAACTTCAACGCCGTGWGGGTGATCGTCGACACCTTGACGAAGGTGGCTCAC  * 58% identity (E-value = 2e-27) with POL_Gypsy of *O. sativa* (AAD04177.1) |
| **>CRM_RNAseH_MG865445**  CCAAATTTTGACAAGATGTTSGAAGTAGAGTGCGACGCTTCATTCATCGGGATTGGAGCTGTCTTATCTCAAGAGAAGAGGCCGGTTGCTTACTTCAGCGAGAAGTTAGGTGGCGCACGCACAAACTATTCGGTGTATGATGTTGAACTCTATGYCATTGTTCAAGCTCTCCAACATTGGCGTCACTATCTCCTCCCAAAAGAGTTTCACCTCTATACCGACCACCAGGCCTTGAAGTTTTTGCAGGATCAAAACAAGCTGAGTGCCCGACNATGCGAAATGG  * 66% identity (E-value = 7e-36) with POL_Gypsy of *A. officinalis* (ABD63193.1) |
| **>Athila_INT_MG865446**  TTTGGTAGCGGKGGATTATGTGTCTAARTGGATTGAGGCCATTGCTTGCCCCAAKTCGGATTCTAAGGTTGTGAAGAAGCTCTTCAAGAAGGTCATATTCCCTCGNGTTTGGTGTACCGAGGGTGGTCATTAGTGATGGMGGTTCTCAYTTYGTCAACCGCACATTAGACAAGCTCTTGGAGAAGTATGGAGTGAGTCACAAGGTGRCCAACGCTTACCATCCACAAACCAATGGGCTAGCCGAGGTGTCAAACCGTGAGGTGAAGCAAATTCTTGAGAAGATGGTAGGTCACTCTAGAAAGGATTGGAGTGACAAGCTTGATGATGCGCTTTGGGCATACCGCACCGCATACAAAACCCCAATTNGGAATGACYCCATTYAAAYTGGTGTATGGGAAACCMTGTCRTCTACCCGTAGAGCTTGAGCATAAGGSATATTGGGCMATC  * 72% identity (E-value = 1e-43) with POL_Gypsy of *A. thaliana* (BAB02143.1) |
| **>Tat_RT_MG865447**  GGATTGAAATTGACCCAAGTAAGATCAAGGCAATCATGGACATGCCTCCTCCCAGAAACCTGAAACAACTGCGTGGTTTACAAGGGCGSTTGGCATACATYCGCCGCTTCATAGCCAAYTTGTCGRGAAAGATYCGTCCGTTCACCCGRCTAACCAAGAAAGAYGTCCCTTTCAAMTGGGAYGGYGAATGCCAAAGYGCWCTYGARGACATCAARGCATATCTCCTCAAACCACCAGTTTTGGCT  * 69% identity (E-value = 7e-30) with POL_Gypsy of *A. officinalis* (ADB63192.1) |

**Table S5.** Blastx alignments using sequences of PCR products compared to GypsyDB cores containing conserved protein regions of plant retrotransposons. Number after NCBI correspond to the PCR products sequence registration number.

_______________________________________________________________________________

Query - Sirevirus_Oryco_integrase (NCBI: MG865448)

Sbjct - Copia_Sirevirus_INT_Vitis (Length=2370)

Score = 190 bits (483), Expect = 2e-56, Method: Compositional matrix adjust.

Identities = 87/139 (63%), Positives = 106/139 (76%), Gaps = 0/139 (0%)

Frame = -1

Query 428 HFISAPRTPQQNGVVERKNRTLQELARTMLNEXSLPTYLWAEAVNTACYVINRVSLRPTL 249

H APRT QQNGVVERKNRTLQE+ARTMLNE +LP Y WAEA+NT+CYV+NR+ LRP L

Sbjct 866 HNFLAPRTSQQNGVVERKNRTLQEMARTMLNENNLPKYFWAEAINTSCYVLNRILLRPIL 925

Query 248 LKTPYELWMGRKPNISYFRAFGSKCFVIDESPKTTKFDSKCIEGIFVGYSLTSKAYRIFI 69

KTPYELW +KPNISYF+ FG KCF+++ KFD+K GIF+GYS +SKA+R+F

Sbjct 926 KKTPYELWKNKKPNISYFKVFGCKCFILNTKDNLGKFDAKSDVGIFLGYSTSSKAFRVFN 985

Query 68 PTSRTIXESIHVXFKENTN 12

+ + ESIHV F E+ N

Sbjct 986 KRTMVVEESIHVIFYESNN 1004

_______________________________________________________________________________

Query - Sirevirus_SIRE_reverse transcripate (NCBI: MG865442)

Sbjct - Copia_Sirevirus_RT_Zea (Length=1553)

Score = 60.5 bits (145), Expect(2) = 7e-20, Method: Composition-based stats.

Identities = 27/49 (55%), Positives = 32/49 (65%), Gaps = 0/49 (0%)

Frame = +1

Query 190 PMXXXXXXDADESGTPVDISQYRAMIGSLLYLTASRSNIMFSVCLCTRF 336

PM D ++ G VD YR+MIGSLLYL ASR +IM SVC+C RF

Sbjct 1311 PMGTDGHIDLNKGGKSVDQKAYRSMIGSLLYLCASRPDIMLSVCMCARF 1359

Score = 49.7 bits (117), Expect(2) = 7e-20, Method: Composition-based stats.

Identities = 20/40 (50%), Positives = 29/40 (73%), Gaps = 0/40 (0%)

Frame = +2

Query 68 TFFLGLQIKQTPEGTFINQAKYAKELIKXFGVEDXXXXXT 187

++FLG Q+KQ +GTFI+Q KY ++LIK FG++D T

Sbjct 1271 SYFLGFQVKQLKDGTFISQTKYTQDLIKRFGMKDAKPAKT 1310

______________________________________________________________________________

Query - Tork_reverse transcripate (NCBI: MG865443)

Sbjct - Copia_Tork_POL_Oryza (Length=2340)

Score = 87.0 bits (214), Expect = 2e-21, Method: Compositional matrix adjust.

Identities = 42/81 (52%), Positives = 55/81 (68%), Gaps = 1/81 (1%)

Frame = -3

Query 240 YEEAIQRDDSKAWQGAMESEIXSMYDNKVWTLVDLPDEKRXIQCKWIFKRKMDLNGN-XS 64

Y EAI DD W AM E+ S+ N W LV LP EK+ I+CKWIFKRK ++ + +

Sbjct 1026 YSEAIVSDDCNRWITAMHDEMESLEKNHTWELVKLPKEKKPIRCKWIFKRKEGISSSDEA 1085

Query 63 TYKARLVAKGFSQIHGIDYDE 1

YKARL+AKG+SQI GID+++

Sbjct 1086 RYKARLIAKGYSQIPGIDFND 1106

_______________________________________________________________________________

Query - Del_integrase (NCBI: MG865444)

Sbjct - Gypsy_Del_Bagy_1_integrase (Length=343)

Score = 105 bits (263), Expect = 3e-29, Method: Compositional matrix adjust.

Identities = 46/83 (55%), Positives = 64/83 (77%), Gaps = 0/83 (0%)

Frame = +1

Query 1 QXLKRHFWWXXMHREVAQYVARCLVCQKVKAERQRPAGLLRPLPKSQRKFDIITMDFVTG 180

Q L++ FWW M RE+A++VA C VC++VKAE QRPAG L+PL + K+D ++MDF+TG

Sbjct 16 QDLRQRFWWTRMKREIAEFVANCDVCRRVKAEHQRPAGTLQPLAIPEWKWDKVSMDFITG 75

Query 181 LPKTQKNFNAVXVIVDTLTKVAH 249

PKT+K NA+ V++D L+KVAH

Sbjct 76 FPKTKKGNNAIFVVIDRLSKVAH 98

_______________________________________________________________________________

Query - CRM_RNase H (NCBI: MG865445)

Sbjct - Gypsy_RNaseH_CRM_Beetle1 (Length=117)

Score = 115 bits (289), Expect = 9e-35, Method: Compositional matrix adjust.

Identities = 54/89 (61%), Positives = 68/89 (76%), Gaps = 0/89 (0%)

Frame = +1

Query 13 KMXEVECDASFIGIGAVLSQEKRPVAYFSEKLGGARTNYSVYDVELYXIVQALQHWRHYL 192

K+ EVECDAS +GIGAVL Q RP+AYFSEKL A+ NYS YD E Y IV+AL +W HYL

Sbjct 1 KVFEVECDASGVGIGAVLQQGGRPIAYFSEKLNHAKLNYSTYDKEFYAIVRALTYWTHYL 60

Query 193 LPKEFHLYTDHQALKFLQDQNKLSARXCE 279

P +F L++DHQALK++ Q+KLS+R +

Sbjct 61 RPAQFVLHSDHQALKYINGQHKLSSRHAK 89

_______________________________________________________________________________

Query - Athila_integrase (NCBI: MG865446)

Sbjct - Gypsy_INT_Athila_Cyclops_2 (Length=338)

Score = 133 bits (334), Expect(2) = 2e-40, Method: Compositional matrix adjust.

Identities = 59/86 (69%), Positives = 71/86 (83%), Gaps = 0/86 (0%)

Frame = +3

Query 108 FGVPRVVISDGGSHFVNRTLDKLLEKYGVSHKVXNAYHPQTNGLAEVSNREVKQILEKMV 287

FG PRV+ISDGGSHF N L+ +L+ YGVSH+V YHPQ NG AEVSNRE+K+ILEK V

Sbjct 113 FGTPRVLISDGGSHFCNAPLESILKHYGVSHRVATPYHPQANGQAEVSNREIKRILEKTV 172

Query 288 GHSRKDWSDKLDDALWAYRTAYKTPI 365

+S+K+WS KLD+ALWAYRTA+K PI

Sbjct 173 SNSKKEWSQKLDEALWAYRTAFKAPI 198

_______________________________________________________________________________

Query - Tat_reverse transcriptase (NCBI: MG865447)

Sbjct - Gypsy_Tat_RT_Asparagus93 (Length=1181)

Score = 125 bits (314), Expect = 5e-35, Method: Composition-based stats.

Identities = 54/80 (68%), Positives = 67/80 (84%), Gaps = 0/80 (0%)

Frame = +3

Query 3 IEIDPSKIKAIMDMPPPRNLKQLRGLQGRLAYIRRFIANLSXKIRPFTRLTKKDVPFXWD 182

I +DP K++AI DMPPP+NLK+LRGLQGRLAYIRRFI+NLS + +PF++L KK + F WD

Sbjct 891 ISLDPEKVRAIQDMPPPKNLKELRGLQGRLAYIRRFISNLSGRCQPFSKLMKKGISFVWD 950

Query 183 GECQSALEDIKAYLLKPPVL 242

GECQ A E+IK YL +PPVL

Sbjct 951 GECQKAFEEIKRYLTQPPVL 970

_______________________________________________________________________________


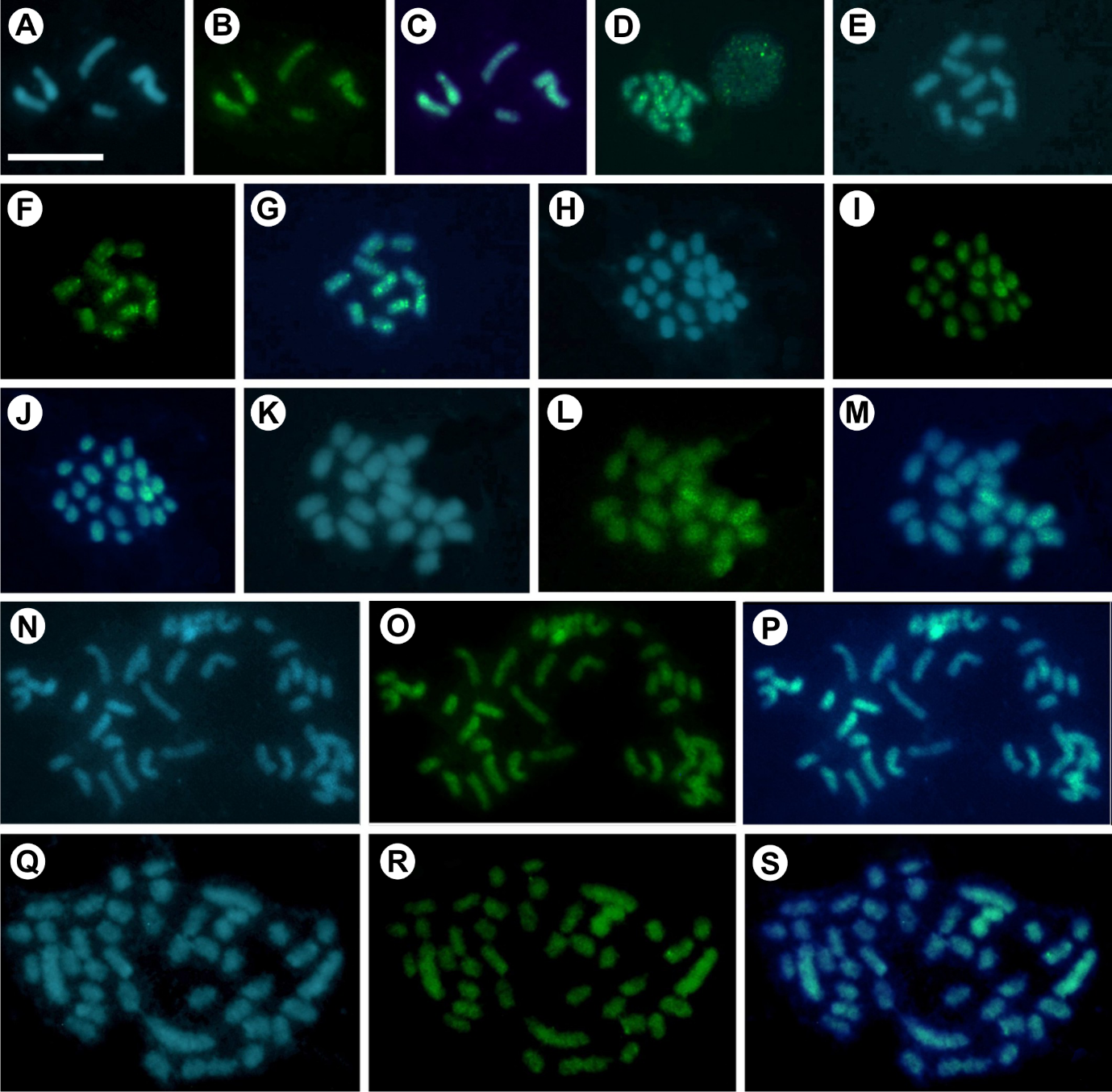
**Figure S4**. FISH using the Oryco (*Copia*) probe on mitotic chromosomes of *Eleocharis*. Chromosomes were DAPI stained (blue) and biotinylated probe was detected with avidin-FITC conjugate (green). Images C, D, G, J, M, P and S are merged. (**A-C**) *E.maculosa* 2*n*=6. Note scattered and very small cluestered signals. (**D-G**) *E. maculosa* 2*n*=10 with more evident clustered signals, including terminal and interstitial ones. Observe in D an interphase nucleus with some regular FISH signals. (**H-J**) *E. geniculata* and (**K-M**) *E. elegans*, both with 2*n*=20, there is a predominance of scattered FISH signals. Note also that half of the chromosome signals are more evident. In *E. montana* (**N-P**) with 2*n*=40 and *E. niederleinii* (**Q-S**) with 2*n*=42, part of chromosomes showed brighter signals, predominantly scattered, but in *E. niederleinii* signals were stronger in three of the four large chromosomes derived from fission and fusion. Bar = 10 μm.

**
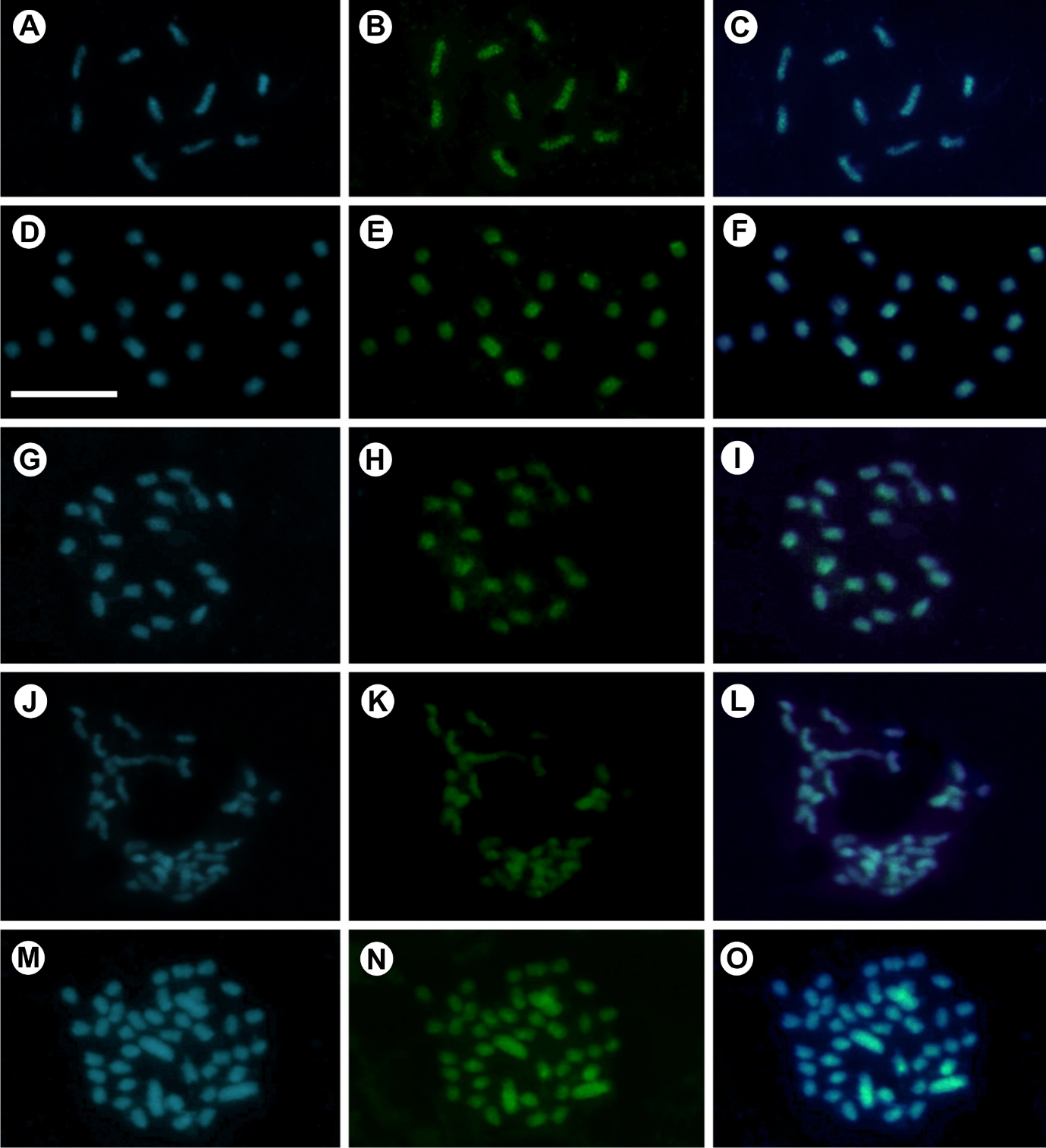
**

**Figure S5.** FISH using the SIRE (*Copia*) probe in mitotic chromosomes of *Eleocharis*. Chromosomes were DAPI stained (blue) and biotinylated probe detected with avidin-FITC conjugate (green). (**A-C**) *E. maculosa* with 2*n* = 10 showing minute clusters along all chromosomes. In *E. elegans* (**D-F**) and *E. sellowiana* (**G-I**), both with 2*n* = 20, scattered signals can be observed in all chromosomes. In at least four chromosomes of *E. elegans* and in the half of chromosomes of *E. sellowiana* signals appeared more intense. Note also some chromosomes with minute clusters (arrows). In *E. montana* (**J-L**) with 2*n*=40 and *E. niederleinii* (**M-O**) with 2*n*=42, part of some chromosomes showed brighter signals, predominantly scattered, but in *E. niederleinii*, the signals were stronger in the four large chromosomes derived from fusion events. Note that these large chromosomes exhibit minute clusters (arrows). Bar = 10 μm.


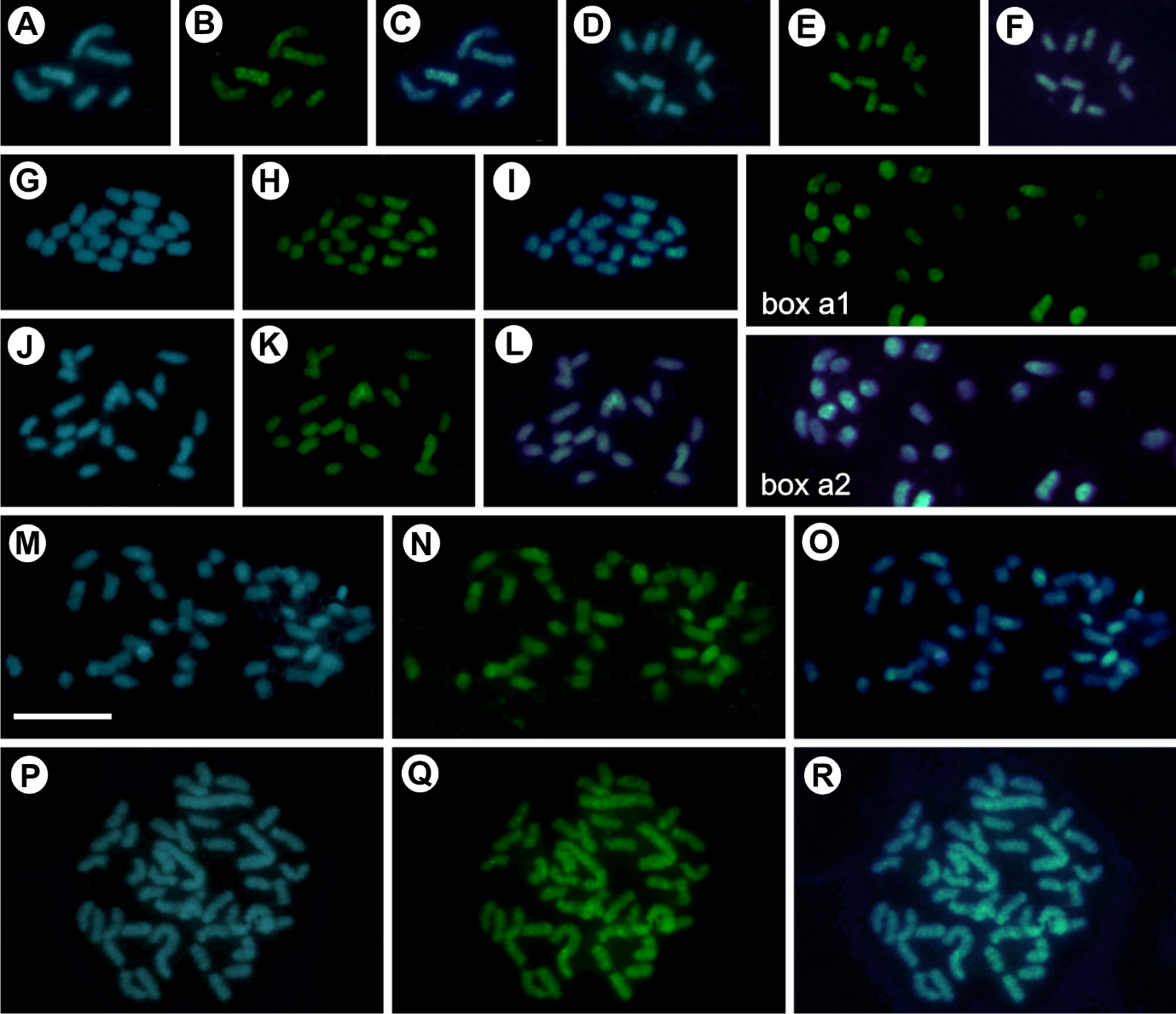


**Figure S6.** FISH using the Tork (*Copia*) probe for mitotic chromosomes of *Eleocharis*. Chromosomes were DAPI stained (blue) and biotinylated probe was detected with avidin-FITC conjugate (green). In *E. maculosa* with 2*n*=6 (**A-C**) and with 2*n*=10 (**D-F**), signals were predominantly scattered, with a pair showing weaker FISH signals (arrows). (**G-I** and **I-L**) In *E. geniculata* and *E. elegans*, both with 2*n*=20, scattered signals were detected in part of the chromosome set, but minute clusters were also observed (arrows). In *E. montana,* with 2*n*=40 (**M-O** and boxes **a1** and **a2**), brighter FISH signals were seen on some or parts of chromosomes, and clusters were more evident (arrows). In *E. niederleinii,* with 2*n*=42 (**P-R**), scattered signals were homogeneously distributed in almost all chromosomes, and clustered signals can be seen in terminal and interstitial regions for some chromosomes (arrows). Bar = 10 μm.


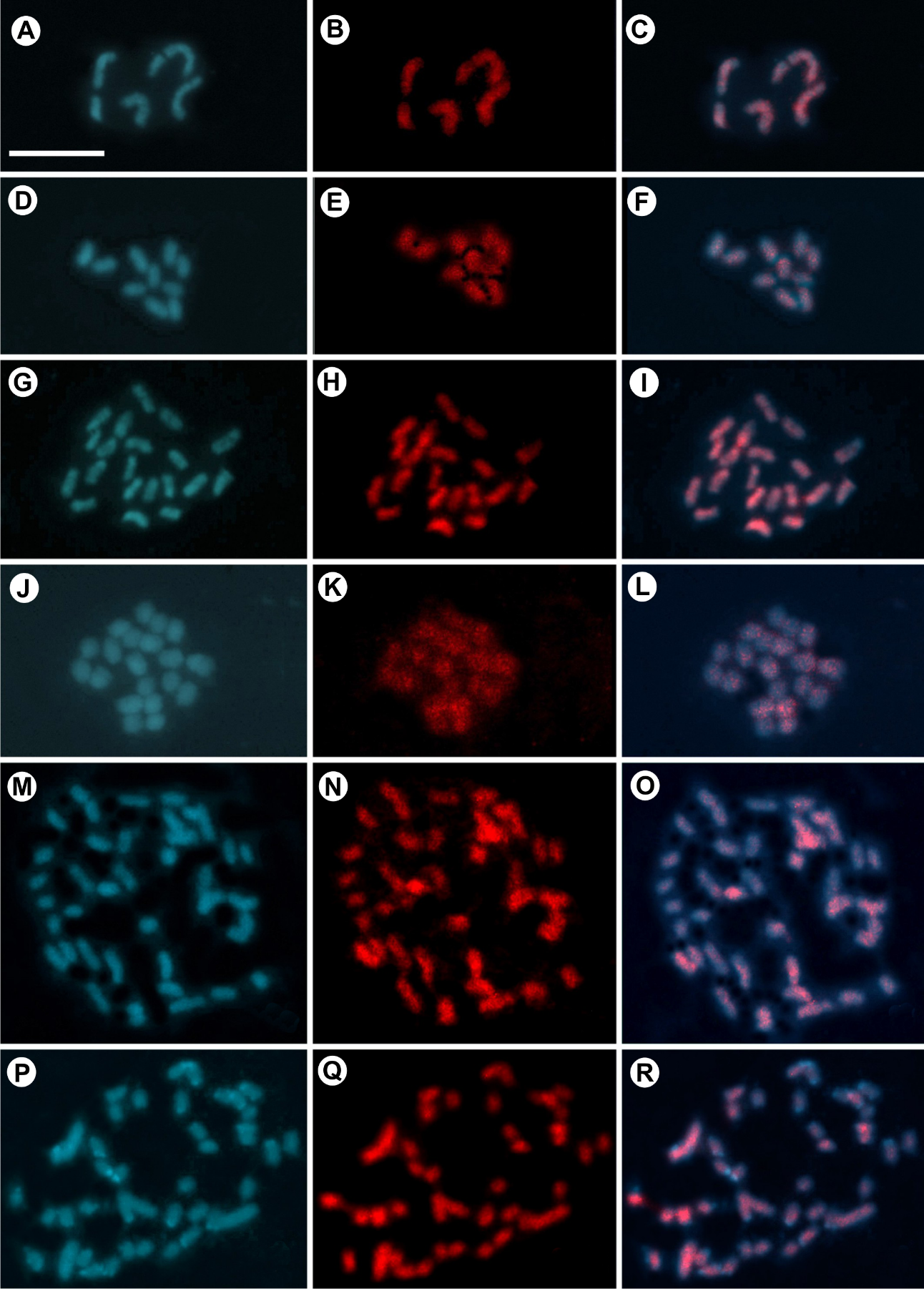


**Figure S7.** FISH using the Del (*Gypsy*) probe in mitotic chromosomes of *Eleocharis*. Chromosomes were DAPI stained (blue) and the Cy3 probe was observed in red. In *E. maculosa,* with 2*n*=6 (**A-C**), the Del probe showed scattered signals distributed along chromosomes, mainly on the four major chromosomes but, in the sample with 2*n*=10 (**D-F**), many small clusters were detected along all chromosomes. (**G-I**) *E. geniculata,* with 2*n*=20, exhibited scattered signals, but more intense signals in six chromosomes. In *E. elegans,* with 2*n*=20 (**J-L),** many small clusters were detected along all chromosomes, with greater abundance on four of them. In *E. montana* with 2*n*=40 (**M-O**) and *E. niederleinii* with 2*n*=42 (**P-R**), FISH signals were more highly accumulated in about 12 and 10 chromosomes (respectively), including both large and small chromosomes. Note that *E. niederleinii* exhibits several terminal AT-rich DAPI-bands, and that this region is poor in LTR-RTs (arrows). Bar = 10 μm.


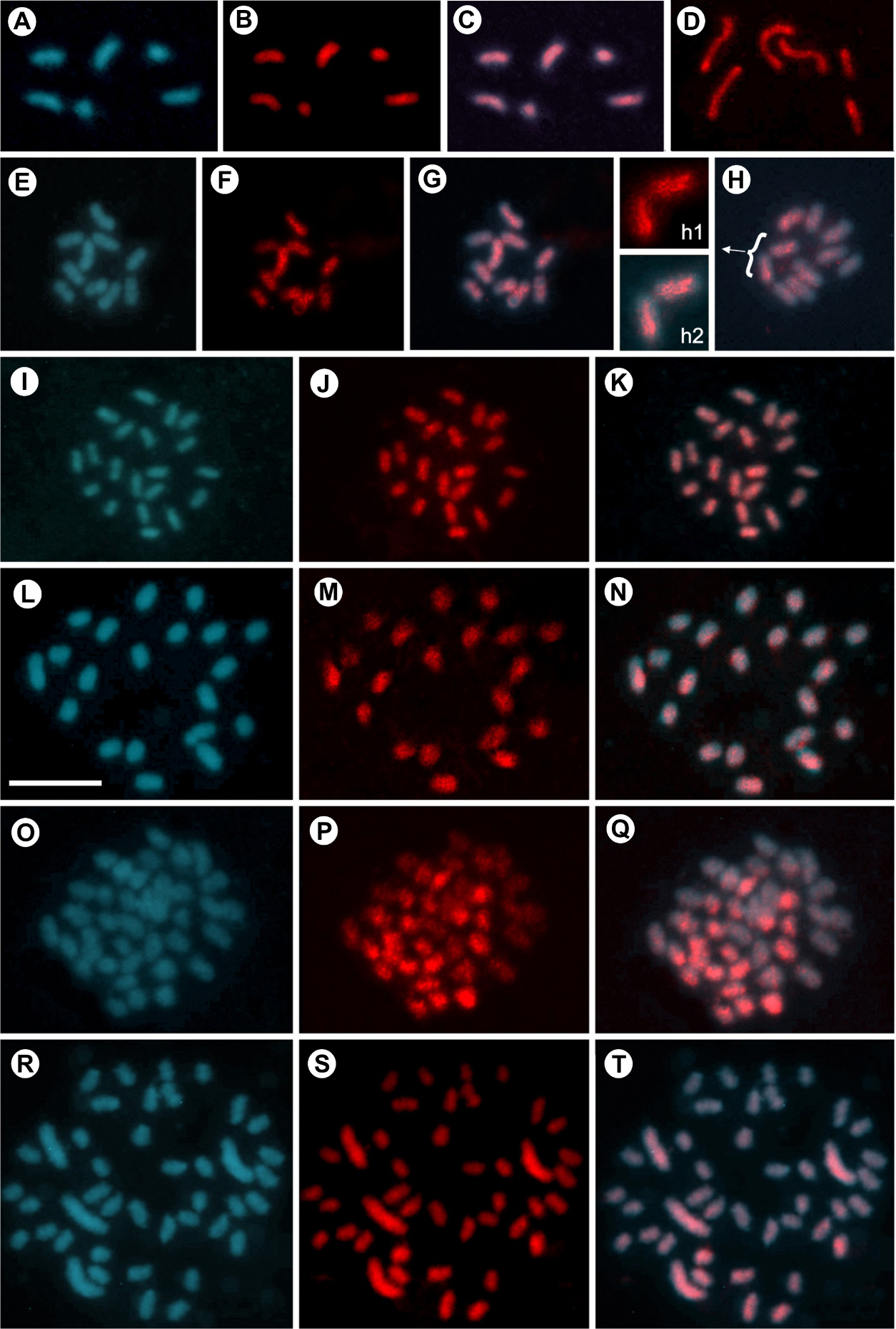


**Figure S8.** FISH using the CRM (*Gypsy*) probe in mitotic chromosomes of *Eleocharis*. Chromosomes were DAPI stained (blue) and the Cy3-labelled probe was observed in red. (**A-C**) *E. maculosa* with 2*n*=6 showed FISH signals distributed along chromosomes, and in prometaphase was possible to note accumulated signals at the chromosome ends (arrows in **D**) besides others small and interstitial. In the sample with 2*n*=10 (**E-H**), scattered and small clustered signals were observed in all chromosomes, but with differential accumulation among them. Note in **H**, **h1** and **h2** an almost linear signal disposition, alike to holocentromeric distribution of CRM/Tyba sat sequences reported by Marques et al. 2015. In *E. geniculata* and *E. elegans*, both with 2*n*=20 (**I-K** and **L-N**), intense FISH signals predominated in half of the chromosomes, beside few small clustered ones (arrows). In *E. montana* with 2*n*=40 (**O-Q**) FISH signals also appeared in all chromosomes, but half of them exhibited a strong accumulation of clustered CRM probe signals. In *E. nierdeleinii* with 2*n*=42 (**R-T**), FISH signals accumulated in 10 chromosomes, including the four large chromosomes derived from fusion events. Bar = 10 μm.


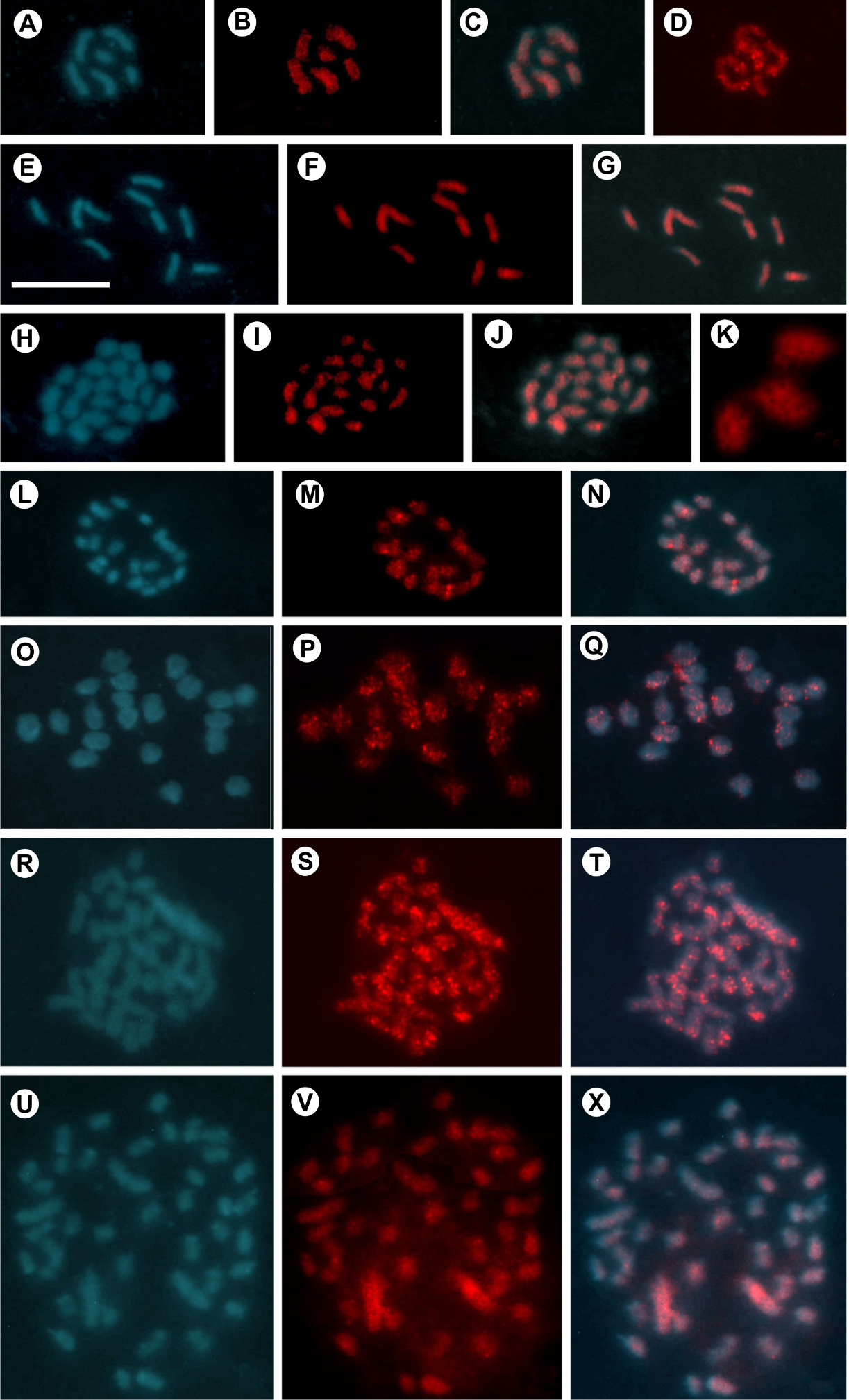


**Figure S9.** FISH using the Athila/Tat (*Gypsy*) probe for mitotic chromosomes of *Eleocharis*. Chromosomes were DAPI stained (blue) and the Cy3 probe was observed in red. In *E. maculosa* with 2*n*=6 (**A-D**) and with 2*n*=10 (**E-G**), FISH exhibited scattered signals along chromosomes, and evident clusters in interstitial and terminal positions (arrows). (**H-K**) *E. geniculata*, with 2*n*=20, exhibited scattered signals (see in **K**) and small clusters that have accumulated in all chromosomes. Note that, in four chromosomes, there seems to be a larger accumulation of Athila/Tat. (**L-N** and **O-Q**) *E. sellowiana* and *E. elegans*, both with 2*n*=20, show a predominance of clustered FISH signals in interstitial and terminal regions. (**R-T**) *E. montana* (2*n*=40) presented a large amount of clustered FISH signals, in all chromosome regions. Arrows point out terminal clusters. In *E. niederleinii,* with 2*n*=42 (**U-X**), finely scattered signals predominated, except for two of the four large and six of the smaller chromosomes. In these two large, one exhibited a continuous marking, while the other showed a cluster in the middle of the chromosome, highlighting the great variability between the chromosomes derived from fission or fusion. Bar = 10 μm.
